# Daily acute intermittent hypoxia elicits age & sex-dependent changes in molecules regulating phrenic motor plasticity

**DOI:** 10.1016/j.expneurol.2025.115240

**Published:** 2025-04-07

**Authors:** Jayakrishnan Nair, Alexandria B. Marciante, Carter Lurk, Mia N. Kelly, Capron Maclain, Gordon S. Mitchell

**Affiliations:** a Breathing Research and Therapeutics Center, Department of Physical Therapy, University of Florida, Gainesville, FL, USA; b Department of Physical Therapy, Thomas Jefferson University, Philadelphia, PA, USA

**Keywords:** Acute intermittent hypoxia, Plasticity, Gene expression, Estradiol, Age, Sex difference, Phrenic long-term facilitation

## Abstract

Acute intermittent hypoxia (AIH) elicits a form of respiratory motor plasticity known as phrenic long-term facilitation (pLTF). Exposure to repetitive daily AIH (dAIH) enhances pLTF, a form of metaplasticity. Little is known concerning cellular mechanisms giving rise to dAIH-induced metaplasticity and the age-dependent sexual dimorphism of AIH associated pro-plasticity mRNA expression. To test if age, sex, and dAIH effects are associated with differential expression of molecules that regulate the Q- and S-pathways and their cross-talk interactions to phrenic motor facilitation, we analyzed key regulatory molecules in ventral spinal (C3-C5) homogenates from young (3-month) and middle-aged (12-month) male and female Sprague-Dawley rats. Since CNS estrogen levels impact molecules known to regulate the Q- and S-pathways, mRNA was correlated with serum estradiol. Rats (*n* = 8/group) were exposed to sham (21 % O2) or dAIH (15, 1 min episodes of 10.5 % inspired O2) per day for 14 days and sacrificed 24 h later. mRNAs for pLTF regulating molecules were assessed via RT-PCR, including: brain-derived neurotrophic factor (*Bdnf*); serotonin 2 A (*Htr2a*), 2B (*Htr2b*), and 7 (*Htr7*) receptors; adenosine 2a (*Adora2a*) receptors; exchange protein activated by cAMP (*Epac1*); p38 MAP kinase [*Mapk14* (α) & *Mapk11* (β)]; PKA regulatory (*Prkar1a*) and; catalytic subunits (*Prkaa1*); fractalkine (*Cx3cl1*), which underlies motor neuron/microglia communication; phosphodiesterase type 4b (*Pde4b*); NAPDH–gp91 (*Cybb*) and p47 (*ncf1*); and the PKC isoform, PKCδ (*Prkcd*). Here we report that age, sex, dAIH preconditioning, and estradiol influence molecules that initiate and/or regulate the Q- and S-pathways to phrenic motor facilitation.

## 1. Introduction

Acute intermittent hypoxia (AIH) persistently increases phrenic nerve activity, an effect known as phrenic long-term facilitation (pLTF). The magnitude of AIH-induced pLTF is enhanced by pre-conditioning with daily AIH (dAIH), or repetitive AIH 3–4 times/week for 4 weeks (Fields and Mitchell, 2015; MacFarlane et al., 2018). Understanding cellular mechanisms and factors regulating dAIH-enhanced plasticity is important since repetitive AIH is emerging as a potential treatment for respiratory and non-respiratory motor deficits with neuromuscular disease or injury (Vose et al., 2022). Based on our nuanced understanding of cellular mechanisms giving rise to pLTF (Mitchell and Baker, 2022), we selected key molecules known to regulate pLTF and investigated dAIH effects on their gene expression (mRNA) in ventral cervical homogenates containing the phrenic motor nuclei. Since age and sex have marked effects on pLTF expression, we investigated age and sex effects on: 1) basal mRNA expression; and 2) dAIH-induced changes in these mRNAs.

**Fig. 1.**
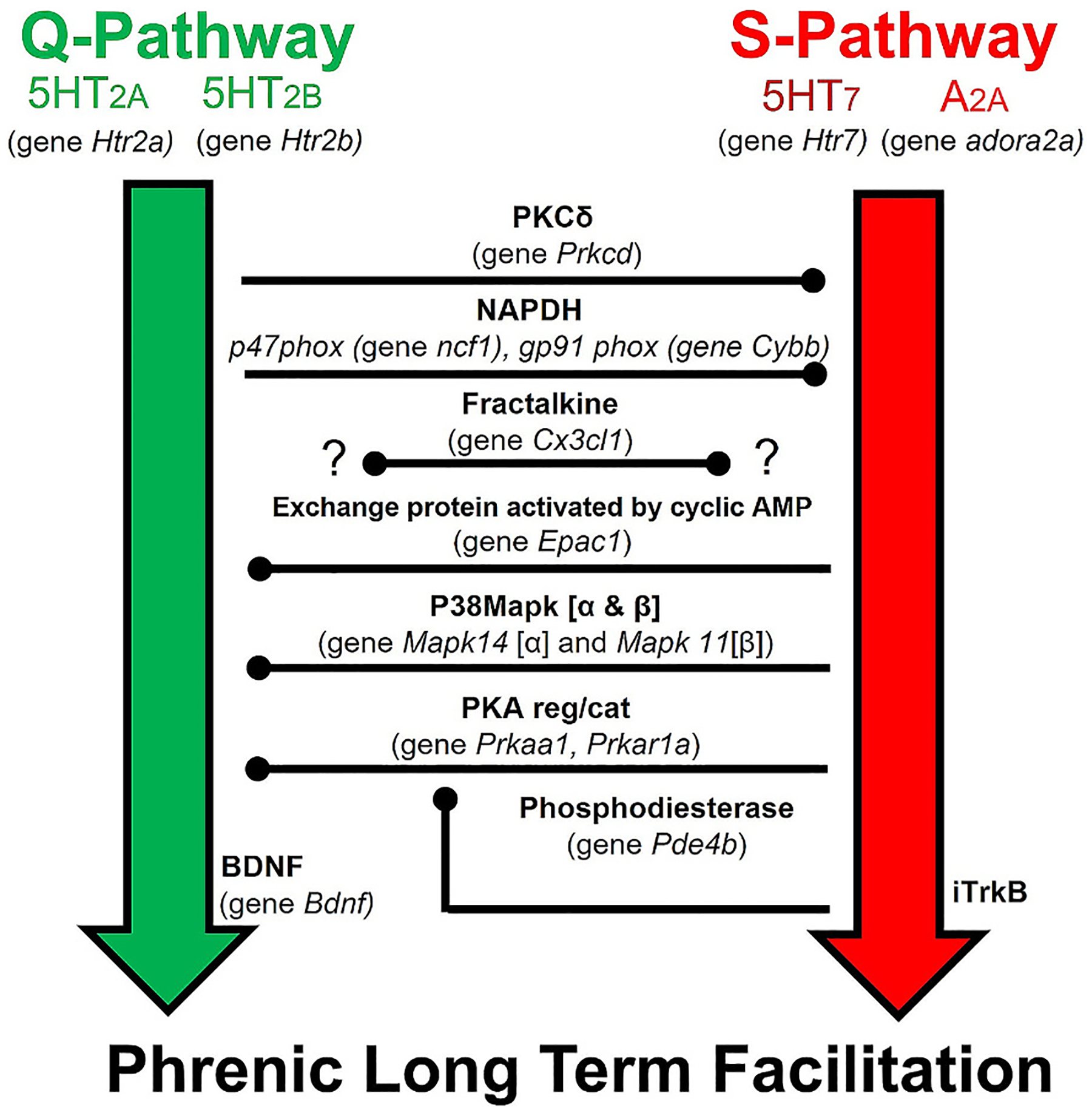
Conceptual framework guiding investigation of intra-ceullular signaling cascades driving or regulating AIH induced phrenic long-term facilitation. Depicted are the Serotonin− 5HT2 A & 5HT2B, BDNF dependent (Q-pathway; green) and the 5HT7 & Adenosine 2 A, immature TrKB (iTrkB) dependent (S-pathway; red). Q to S-pathway cross talk inhibition is mediated by PKCδ or NADPH (p47 and gp91 are key subunit genes). S to Q-pathway cross talk inhibition is mediated by Protein Kinase A (PKA; key subunit genes encode regulatory and catalytic domains), with suspected involvement of p38Map kinase. Phosphodiesterase indirectly inhibits PKA activity by decreasing cAMP levels, liberating the Q-pathway and suppressing S-pathway activation. Fractalkine protein activates its receptor on nearby microglia, triggering them to increase extracellular adenosine, thus suppressing the Q-pathway and favoring the S-pathway. Genes associated with each of these molecules are shown in parenthesis.

Activation or inhibition of phrenic motor neuron Gq (Q-pathway) or Gs (S-pathway) protein coupled receptors guided studies of cellular mechanisms giving rise to and regulating plasticity (Dale-Nagle et al., 2010; Mitchell and Baker, 2022). With moderate AIH (PaO_2_ ≳ 40 mmHg), Gq-coupled serotonin (5HT2) receptor-dependent pLTF predominates (Baker-Herman and Mitchell, 2002; Fuller et al., 2001; Kinkead et al., 1998; MacFarlane et al., 2008). This Q-pathway driven plasticity requires downstream ERK MAP kinase, TrkB, and protein kinase C-θ (PKCθ) activity (Dale et al., 2017; Devinney et al., 2015; Hoffman et al., 2012), new BDNF protein synthesis (Baker-Herman and Mitchell, 2002), reactive oxygen species (ROS) formation (MacFarlane et al., 2009; MacFarlane et al., 2008), and neuronal nitric oxide synthase (nNOS) activity (MacFarlane et al., 2014). Conversely, with severe AIH (PaO_2_ ≲ 30 mmHg), pLTF is driven by GS-coupled adenosine 2 A (A2A) and 5HT7 receptor activation (Golder et al., 2008; Hoffman and Mitchell, 2011; Nichols et al., 2012; Nichols and Mitchell, 2021). S-pathway signaling requires new synthesis of an immature TrkB isoform (vs BDNF) and PI3 kinase/Akt signaling (vs ERK) (Golder et al., 2008). Remarkably, with PaO_2_ between ~30–35 mmHg during AIH, Q-S pathway co-activation cancels pLTF due to powerful cross-talk inhibition (Fig. 1) (Perim and Mitchell, 2019).

AIH protocols consisting of shorter hypoxic episodes minimize hypoxia-evoked spinal adenosine accumulation and S-Q cross-talk inhibition (Marciante et al., 2023). However, multiple conditions shift the serotonin/adenosine balance (Mitchell and Baker, 2022). For example, with spinal cord injury, heightened tissue hypoxia from systemic hypotension and/or pericyte capillary constriction (Li et al., 2017) increase adenosine accumulation and Q-S cross-talk inhibition at the same arterial PO_2_ (Perim et al., 2021a; Perim et al., 2023). Time of day (Marciante et al., 2023), age and sex (Behan et al., 2002; Behan et al., 2003; Dougherty et al., 2017; Zabka et al., 2001a, 2001b) also regulate pLTF due to shifts in the serotonin/adenosine balance (Marciante and Mitchell, 2023b; Marciante et al., 2023). Phrenic LTF elicited by AIH consisting of 1 min hypoxic episodes is attenuated in the rodent active (vs rest) phase due to diurnal variations in spinal adenosine (Marciante et al., 2023). Rest phase pLTF decreases with age in males, but increases in middle-aged females, and exhibits pronounced estrus cycle fluctuations; these effects may arise from estradiol effects on the Q- or S-pathways and their cross-talk interactions (Dougherty et al., 2017; Zabka et al., 2006).

Pretreatment with daily AIH (dAIH) enhances pLTF, demonstrating metaplasticity (Fields and Mitchell, 2015; Mitchell and Johnson, 2003). Although mechanisms whereby dAIH enhances pLTF are unknown, they may relate to: 1) Q-pathway amplification (MacFarlane et al., 2018; Satriotomo et al., 2012); and/or 2) minimizing S-Q cross-talk constraints (Hoffman et al., 2010; Mitchell and Baker, 2022). Since aging increases S-to-Q cross-talk inhibition via increased spinal adenosine levels in geriatric male and female rats (Marciante and Mitchell, 2023a), differential dAIH effects with age and sex must be considered. Since pLTF is impacted by the female estrus cycle, it is important to account for estrogen effects on the Q- and S-pathways (Dougherty et al., 2017; Zabka et al., 2006). Here, we begin exploring these relationships by screening mRNAs for molecules that initiate or regulate pLTF in ventral cervical homogenates with and without dAIH in young and middle-aged male and female rats.

We hypothesize that 2 weeks of dAIH: 1) decreases ventral cervical spinal mRNA of molecules mediating Q- and S-pathway cross-talk inhibition; and/or 2) increases mRNA of key Q-pathway molecules. We further hypothesize that age and sex regulate mRNA of molecules known to regulate pLTF, and that these effects correlate with serum estradiol.

## 2. Materials and methods

### 2.1. Animals

All experimental procedures were approved by the Institutional Animal Care and Use Committee at the University of Florida and conformed to policies detailed in the National Institutes of Health Guide for the Care and Use of Laboratory Animals. Experiments were performed on naïve 3-month-old male (297 ± 16 g, *n* = 8) and female (243 ± 7 g, n = 8), and on naïve 12-month-old, middle-aged, male (493 ± 16 g, n = 8) and female (286 ± 55 g, n = 8) Sprague Dawley rats (208 A Colony, Inotive formally known as Envigo, IN). Rats were housed in pairs in individually ventilated cages and maintained in a mixed-sex rodent room within an AAALAC-accredited animal facility for at least one week prior to use. The ambient conditions at the facilities were maintained at 24 °C with 12 h light-dark cycles (6 am- 6 pm) with access to food and water ad libitum. Sample size estimation was based on prior studies (McIntosh and Dougherty, 2019; Zabka et al., 2001b).

### 2.2. Daily AIH protocol

**Fig. 2.**
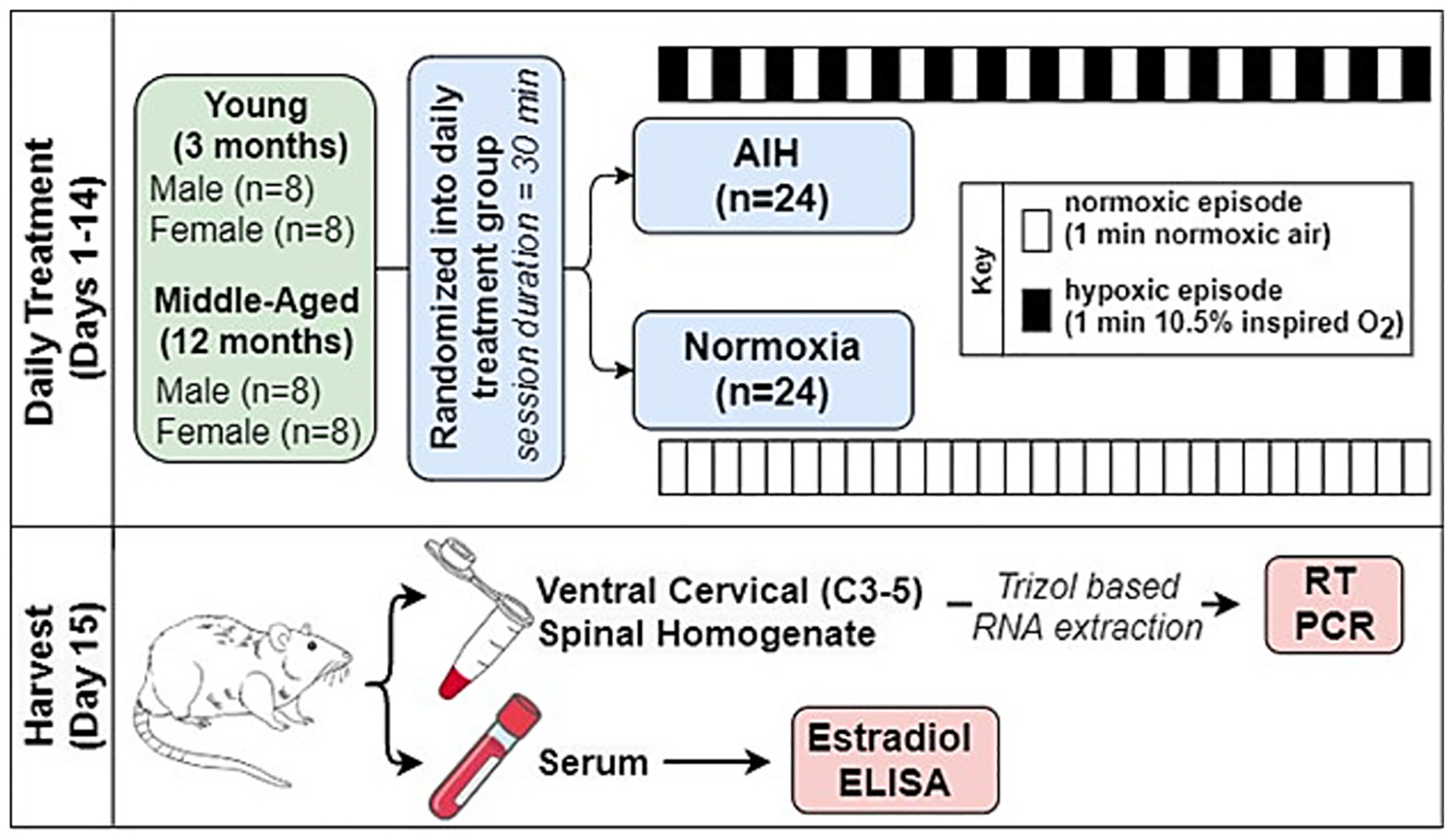
Schematic of the experimental design. Young [3 months, *n* = 12 (male = 6; female = 6)] and middle aged [12 months, n = 12 (male = 6; female = 6)] rats of both sexes were exposed to daily AIH for 14 consecutive days (15, 1 min hypoxic episodes with 1 ½ min normoxic intervals) and equal number of age and sex matched rats (*n* = 24) were exposed to Sham (normoxia). 24 h after the last exposure, rats were sacrificed under deep isoflurane anesthesia and ventral cervical spinal cord tissues were dissected and flash frozen for mRNA analysis using RT PCR. At the same time serum samples from these rats were collected and stored for estradiol ELISA.

Rats were randomly assigned to receive either daily poikilocapnic AIH (dAIH) or continuous normoxia (Sham) exposures. dAIH consisted of 15, 1-min hypoxic episodes (10.5 % inspired O2) separated by 1-min normoxic intervals; AIH was repeated daily for 14 consecutive days (Fig. 2). On exposure days, rats were placed in individual, custom-designed cylindrical exposure chambers (volume: 1 L) with flat inserts to support the rats and enable urine to pass below in the platform. Seven chambers were parallelly connected to a computer-controlled programmable system of mass flow controllers (Flow Commander, Therapeutiq Research, Kansas, USA) to regulate chamber gas flow (6 L/min, randomly sampled from one of the sampling chambers) and gas composition, with targeted inspired O_2_ levels (Fig. 1). Cotton gauze placed close to the airflow inlet dispersed incurrent gas flow. This system allowed automated cycling between hypoxia and room air or sham normoxia. Rats were allowed ~30 min to acclimate to the chamber before AIH began. Exposures were given between 8:30–11 AM each day, and the rats were then returned to their home cages.

### 2.3. Necropsy and sample collection

24 h after dAIH, rats were euthanized under deep isoflurane anesthesia (4 %) and decapitated via rodent guillotine. The 24-h time point was chosen to avoid the confounding immediate effects of AIH and quantify only the true metaplasticity effects on gene expression. Prior to sacrifice, the estrus cycle of female rats was determined via vaginal swabs as described by others (Marcondes et al., 2002). Blood was collected in a serum separator vial and allowed to clot for 20–30 min at room temperature. The sample was centrifuged at 3000 RPM for 20 min. Separated serum was removed and stored at −80 °C for ELISA-based estradiol analysis. Following decapitation, C3-C5 spinal segments were rapidly removed and immersed in ice-cold, sterile phosphate buffer solution (PBS). Spinal cord samples were transferred to a freezing microtome (−22 °C), cut at the level of the central canal with a double edge blade, and saved in RNA later stabilization solution (Thermo Fisher Scientific); these maneuvers were achieved within 14 ± 1.1 min from the time of sacrifice. Ventral C3-C5 was placed in RNA later stabilization solution and stored at 4 °C overnight before transferring to −80 °C until further processing (i.e. 1–3 months). On the RNA isolation day, frozen samples were thawed on ice and wet weights were recorded (38 ± 5.6 g; mean ± SD).

### 2.4. RNA isolation and quantification procedures

Tissue samples were homogenized in 800 μl TRIzol Reagent (Invitrogen) using Bead Mill 4 Homogenizer (Thermo Fisher Scientific). The extract was cleaned by ethanol precipitation and transferred to the RNeasy spin column (RNeasy kit, Qiagen), and purified. RNA concentration was quantified via spectrophotometry (ʎ = 260 nm; NanoDrop model 2000C, ThermoFisher Scientific). RNA purity was estimated by the absorbance ratio A_260_/A_280_ (all samples had a ratio from 1.8 to 2.0).

### 2.5. RT-PCR

Extraction and quantification procedures as described in earlier publication (Kelly et al., 2020) were used for RT-PCR. The first strand complementary DNA (cDNA) was synthesized from 2.5 μg of total RNA using random primers in SuperScript VILO cDNA Synthesis Kit (Invitrogen). Resulting cDNAs were diluted to 4 ng/μL and used as templates in real-time quantitative polymerase chain reactions (PCRs; Quant-Studio3; Applied Biosystems). The following genes were analyzed in this study: brain derived neurotrophic factor (gene:-*Bdnf*); serotonin 2 A (*Htr2a*), 2B (*Htr2b*), and 7 (*Htr7*) receptors; adenosine 2a (*Adora2a*) receptors; exchange protein activated by cAMP (*Epac1*); p38 MAP kinase [*Mapk14* (α) & *Mapk11* (β)]; PKA regulatory (*Prkar1a*) and; catalytic subunits (*Prkaa1*); fractalkine (*Cx3cl1*), which underlies motor neuron/microglia communication; phosphodiesterase type 4b (*Pde4b*); NAPDH–gp91 (*Cybb*) and p47 (*ncf1*); and the PKC isoform, PKCδ (*Prkcd*). TaqMan oligonucleotide primers and probe sets (TaqMan Gene Expression Assays; Applied Biosystems) used for PCR are listed in Table 1.

A mastermix was prepared for each investigated gene and all the groups were compared using the same mix. Twenty μL reaction mix was prepared for each PCR well. Reaction mixtures consisted of 5 μL cDNA (4 ng/μL), 10 μL TaqMan Fast Advanced Master Mix (Applied Biosystems), 4 μL DEPC-treated RNAse-free water, and 1 μL of the corresponding TaqMan gene expression assay. Taq-Man primers and probes were used in duplicate reactions, with the same cycling conditions (50 °C for 2 min, and 95 °C for 2 min, followed by 40 cycles of 95 °C for 1 s and 60 °C for 20 s). Each RTPCR plate contained cDNA samples and their negative control reactions containing random primers. Negative control reactions confirmed minimal contamination. mRNA in ventral C3–C5 homogenates were normalized as a difference from the amplification cycle at the 18 s mRNA threshold. Quantification was performed via the ΔΔCT method (Livak and Schmittgen, 2001); ΔCT values were calculated as CT (target gene) - CT (18 s). mRNA levels are presented as fold change (2^–ΔΔCT^) from young males exposed to sham normoxia.

### 2.6. Estradiol ELISA

**Fig. 3.**
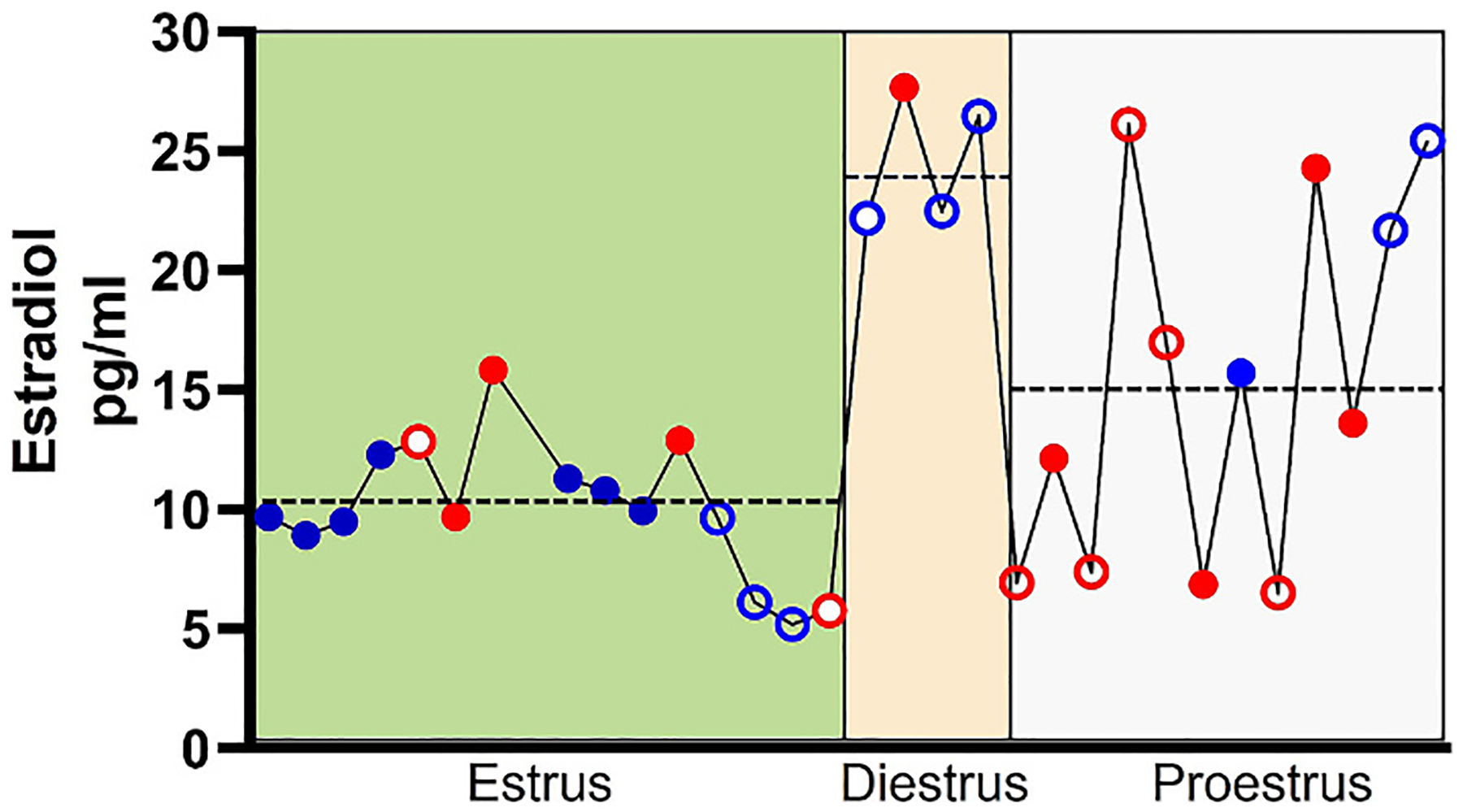
Correlation between serum estradiol and estrus cycle stage. Correlation between serum estradiol and estrus cycle stage. The estrus cycle for female rats was determined via vaginal swab smear. The serum estrodial level was lower during estrus (m ± SD; 10.0 ± 2. 8 pg/ml) and elevated during diestrus (24.7 ± 2.4 pg/ml). Large variability characterized proestrus (15.3 ± 7.3 pg/ml). Dotted line = mean for that stage. dAIH exposed animals were disproportionately in the proestrus phase (9/15 = 60 %). *Open circles* = *Younger; Closed Circles* = *Middle aged; Blue* = *Normoxia; Red* = *dAIH*.

Frozen serum samples were thawed on ice and estradiol levels assayed via competitive immunoassay in rat serum (BioVendor, Cat#RTC009R) (Marciante and Mitchell, 2023a). The calibration range for the assay was 2.5–1280 pg/ml. The batch assay was performed according to the manufacturer’s instructions on all the sample concurrently. A filter-based, single-channel absorbance (range 400–750 nm) microplate reader (BioTek 800 TS) was used to quantify the level of serum estradiol level. The correlation between serum estradiol and estrus cycle is presented in Fig. 3. The estrus cycle, as determined with vaginal swab smear shows that the serum estradiol was lower in estrus (10.0 ± 2.8 pg/ml) and higher during diestrus phase (24.7 ± 2.4 pg/ml). Estradiol exhibited a high degree of variability in proestrus (15.3 ± 7.3 pg/ml; all values mean ± S.D.). Additionally, we also observed that dAIH exposed female rats were disproportionately in the proestrus phase (*n* = 9/15 = 60 %). However, overall, estradiol levels were not significantly different between exposure or age groups.

### 2.7. Statistical analysis

To study the effects of age (3 vs 12 months), sex (male vs female) and treatment (dAIH vs Sham) on gene expression (dependent variable), a 3 way ANOVA with all interactions used for each outcome (SPSS). Univariate main effects are expressed in mean difference (MD), with F and *p* values provided. Interactions effects are expressed in parameter estimates (β), F and p values. A post-hoc analysis with Bonferroni correction was performed for all possible pairwise comparisons. The effect of treatment within groups defined by age and sex, age effect defined by groups of sex and treatment, and effects of sex defined by groups of age and treatment. To account for multiple comparisons, we used a conservative error rate (α) of *p* ≤ 0.01 to determine significance for age and sex effects, and sham vs dAIH treatment. The correlation between estradiol and gene expression was assessed via simple linear regression (Prism Graph Pad). Significant differences in slope (α error of p ≤ 0.01) between Sham and dAIH-exposed rats was used to determine possible effects of estradiol on dAIH-induced changes in gene expression. Outlier data points were removed from the statistical analysis if the Cook’s D was >4. Values are means ± SD.

### 2.8. Elimination criterion

Each cohort had 8 rats, although data from 2 rats in the young male sham cohort were not included since the 18 s reference gene values had a Cook’s D > 4. Values from one middle-aged rat for fractalkine and 5HT7 gene expression were removed from the analysis as these data points had a Cook’s D value >4. Serum estradiol values from 1 young female and 2 middle-aged males could not be collected due to a sample processing error.

## 3. Results

### 3.1. Effect of age, sex and daily AIH (dAIH) on genes associated with receptors driving the Q and S-pathways

**Fig. 4.**
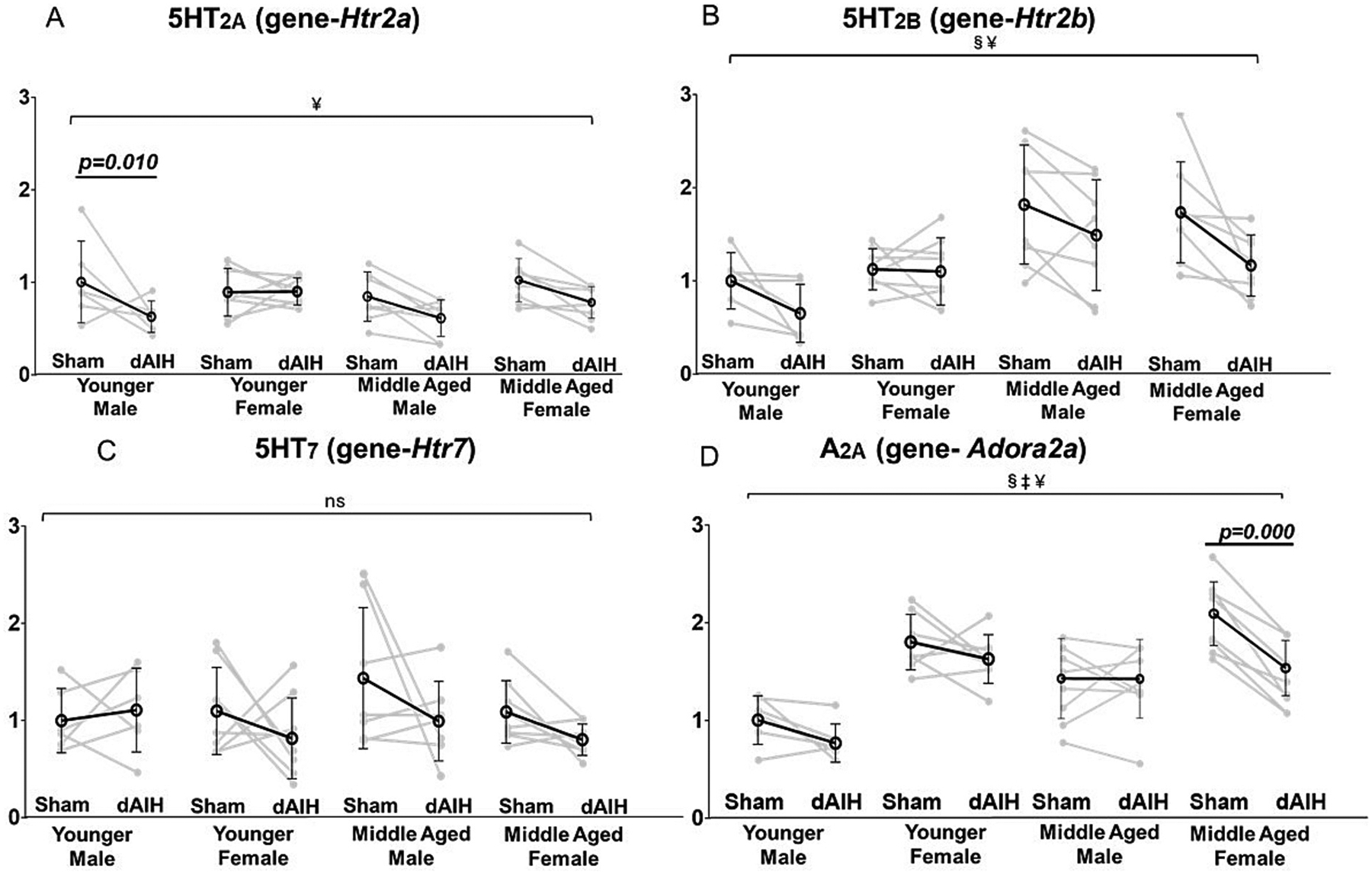
Effect of age, sex and daily AIH (dAIH) on genes associated with receptors driving the Q and S-pathways: 5HT2A (*Htr2a*), 5HT2B (*Htr2b*), 5HT7 (*Htr7*), and A2A (*Adora2a*). A significant main effect of dAIH treatment was observed on 5HT2A expression, with a significant reduction of receptor expression in younger male (Panel A). A significant main effect of age and dAIH was observed on 5HT2B receptor expression (Panel B). 5HT2B expression is downregulated by dAIH exposure. A significant main effect of age, sex, and treatment was observed with A2A expression. A2A expression was reduced with dAIH exposure in middle-aged females (Panel D). No main effect of age or sex was observed in 5HT2A and 5HT7 expression (Panel A, C), and none of the Q & S receptor expressions had a significant interaction effect of age, sex, or treatment. §-Significant main effect of age; ‡ − Significant main effect of sex; ¥ - Significant main effect of treatment.

A statistically significant increase in A2A (MD = 0.27, F = 10.19, *p* = 0.002, Fig. 4D), and 5HT2B (MD = 0.579, F = 24.81, *p* < 0.001, Fig. 4B) receptor mRNA expression was observed with age in the ventral cervical spinal cord (Table 2). On pairwise comparisons, A2A receptor increases were detected in dAIH-exposed middle aged male rats (young = 0.76 ± 0.20; middle-aged = 1.36 ± 0.39; *p* = 0.001). 5HT2B receptor expression increased in sham-exposed middle aged males (young = 1.00 ± 0.30; middle-aged = 1.82 ± 0.64; *p* = 0.001) and middle aged females (young = 1.13 ± 0.22; middle-aged = 1.73 ± 0.54; *p* = 0.009), demonstrating a shift with increasing age unrelated to dAIH. However, middle aged males also exhibited increased 5HT2B expression in response to dAIH (young = 0.65 ± 0.31; middle-aged = 1.49 ± 0.60; p = 0.001; Table 3).

An overall main effect of sex on A2A receptor was observed with significantly higher mRNA level in younger and middle-aged females (MD = 0.60, F = 52.04, *p* < 0.001, Fig. 4D; Table 2). Pairwise comparisons demonstrate greater A2A mRNA in sham-exposed young females (male = 1.00 ± 0.25; female = 1.79 ± 0.28; *p* < 0.001) and middle-aged females (male = 1.37 ± 0.39; female = 1.6 ± 0.38; p < 0.001). dAIH-exposed young females also exhibit higher A2A receptor expression (young male = 0.76 ± 0.20; young female = 1.62 ± 0.25; p < 0.001). No sex-specific differences in A2A receptor expression were observed in middle-aged rats exposed to dAIH (middle-aged male = 1.36 ± 0.39; middle-aged female = 1.07 ± 0.33; *p* = 0.696; Table 4).

An overall main effect of dAIH was detected in mRNA levels for 5HT2A (MD = −0.21, F = 11.06, p = 0.002, Fig. 4A), 5HT2B (MD = −0.39, F = 7.48, *p* = 0.009, Fig. 4B), and A2A receptors (MD = −0.27, F = 10.4, *p* = 0.003; Fig. 4D, Table 2). However, dAIH only reduced expression of 5HT2A receptor mRNA in younger males (Sham = 1.00 ± 0.44; dAIH = 0.63 ± 0.17; *p* = 0.010, Fig. 4A) and A2A receptor mRNA in middle-aged females (Sham = 1.6 ± 0.38; dAIH = 1.07 ± 0.33; *p* = 0.000, Fig. 4D). No significant effect of age, sex, or dAIH treatment was observed on 5HT7 mRNA expression (Fig. 4C; Tables 3, 4 & 5).

### 3.2. Effect of age, sex and daily AIH (dAIH) on genes associated with Q to S cross-talk inhibition

Overall, age was associated with increased Q to S crosstalk molecules, including p47 (MD = 0.59, F = 35.72, p = 0.001) and gp91 (MD = 0.48, F = 17.50, p = 0.001); and no significant association with PKCδ expression (MD = 4.92, F = 0.74, *p* = 0.031; Table 2). However, pairwise comparisons reveal significant age effects, with higher PKCδ (young = 1.00 ± 0.28; middle-aged = 1.60 ± 0.69; *p* = 0.008) and p47 expression (young = 1.00 ± 0.25; middle-aged = 1.80 ± 0.59; *p* < 0.001) in sham exposed middle-aged males. With dAIH, middle-aged males had increased PKCδ (young = 0.62 ± 0.03; middle-aged = 1.28 ± 0.13; *p* = 0.005) and p47 expression (young = 0.80 ± 0.23; middle-aged = 1.40 ± 0.44; *p* = 0.003). In contrast, PKCδ levels was significantly reduced in dAIH exposed middle-aged females (young = 1.57 ± 0.52; middle-aged = 0.8 ± 0.17; *p* < 0.001; Table 3).

Sex has a significant overall effects on PKCδ (MD = 0.31, F = 8.09, *p* = 0.006) and p47 expression (MD = 0.26, F = 7.19, p = 0.010). Pairwise comparisons revealed a significant elevation in PKCδ mRNA in dAIH exposed young female vs male rats (young male = 0.62 ± 0.03; young female = 1.57 ± 0.52; *p* < 0.001; Table 4).

**Fig. 5.**
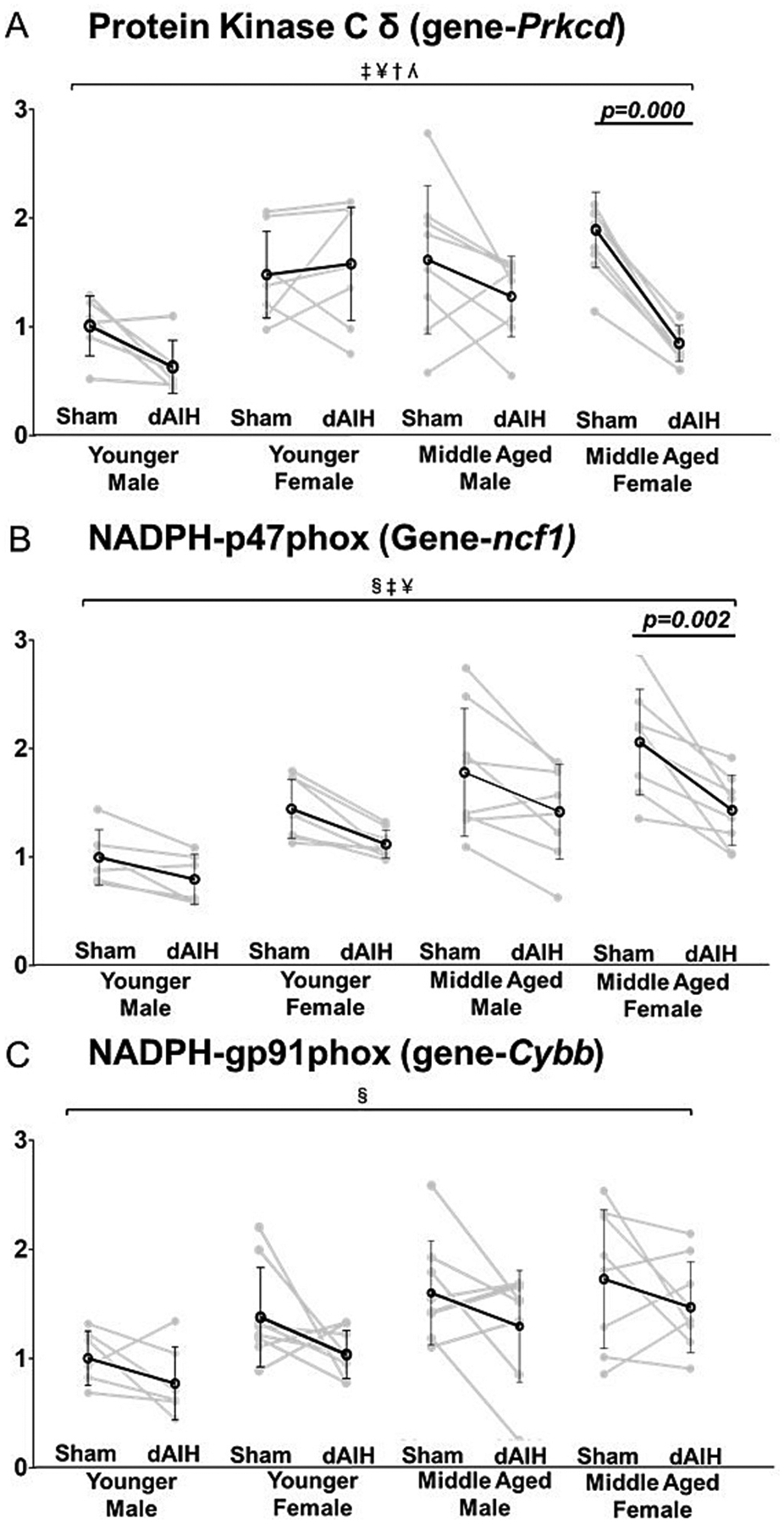
Effect of age, sex and daily AIH (dAIH) on genes associated with Q to S cross talk inhibition: PKCδ (*Prkcd*), NAPDH-p47 (*Ncf1*), and NAPDH-gp91 (*Cybb*). A significant main effect of sex and treatment was observed in PKCδ expression, with a significant age-sex and age-sex-dAIH treatment interaction (Panel A). dAIH treatment significantly reduced PKCδ expression only in middle-aged females (Panel A). A significant main effect of age, sex, and treatment was observed in NADPH-p47 expression. Exposure to dAIH treatment significantly reduced NADPH-p47 expression in middle-aged females (Panel B). A significant main effect of age was observed with NADPH-gp91 expression (Panel C). None of the interactions were significant for NADPH-p47 or gp91. §-Significant main effect of age; ‡ − Significant main effect of sex; ¥ - Significant main effect of treatment; † − Significant age*sex interaction; ʎ - Significant age*sex*treatment interaction.

An overall main effect of dAIH was detected in mRNAs of PKCδ (MD = −0.42, F = 14.74, *p* < 0.001) and p47 (MD = −0.39, F = 15.04, p < 0.001; see Table 2). A significant interaction effect of age × sex (β = −1.40, F = 13.73, p < 0.001), and age × sex × dAIH (β = 1.18, F = 7.46, p = 0.009) was observed in PKCδ expression (Table 2). dAIH effects on Q to S cross-talk molecules was primarily driven by changes in middle aged females as evident from a significant reduction of PKCδ mRNA (Sham = 1.89 ± 0.35; dAIH = 0.8 ± 0.17; p = 0.000) (Fig. 5A), and p47 mRNA expression (Sham = 1.94 ± 0.55; dAIH = 1.35 ± 0.34; *p* = 0.002) (Fig. 5B; Table 5).

### 3.3. Effect of age, sex and daily AIH (dAIH) on genes associated with S to Q cross-talk

An age associated overall decrease in phosphodiesterase-4b mRNA (MD = −0.16, F = 9.45, p = 0.003; Table 2) was observed. Pairwise comparisons indicate that phosphodiesterase-4b expression decreases with age in sham-exposed females (young female = 1.42 ± 0.19; middle-aged female = 1.07 ± 0.22; *p* < 0.001) and dAIH-exposed females (young female = 1.09 ± 0.14; middle-aged female = 0.78 ± 0.16; *p* < 0.003). In contrast, no significant age associated changes were observed in males (Table 3).

Overall females expressed greater levels of phosphodiesterase-4b (MD = 0.22, F = 18.26, p < 0.001), p38MAPkinase β subunit (MD = 0.49, F = 34.98, p < 0.001), p38MAPkinase α subunit (MD = 0.29, F = 10.18, *p* = 0.002), PKA regulatory subunit (MD = 0.36, F = 27.63, p < 0.001), and PKA catalytic subunit (MD = 0.33, F = 30.32, p < 0.001) versus age matched males. Significant interactions of age × sex were detected in mRNA for p38MAP kinase β subunit (β = −0.31, F = 8.44, *p* = 0.005), and phosphodiesterase-4b (β = −0.44, F = 11.32, *p* = 0.001; Table 2).

Pairwise comparisons indicated higher phosphodiesterase-4b mRNA expression in young females exposed to sham (male = 1.00 ± 0.23; female = 1.42 ± 0.19; p < 0.001) and dAIH (male = 0.73 ± 0.16; female = 1.09 ± 0.14; p = 0.001). p38 MAP kinase β subunit mRNA expression was also higher in young females exposed to sham (male = 1.08 ± 1.17; female = 1.86 ± 0.37; p < 0.001) and dAIH (male = 0.66 ± 1.39; female = 1.36 ± 0.24; p < 0.001). Finally, p38 MAP kinase α subunit mRNA expression was greater in young females exposed to sham (male = 1.04 ± 1.24; female = 1.68 ± 0.45; *p* < 0.001; Table 4) but not in dAIH (male = 0.93 ± 1.24; female = 1.24 ± 0.31; *p* = 0.310).

PKA regulatory subunit mRNA expression was higher in young females exposed to sham (male = 1.00 ± 0.21; female = 1.60 ± 0.39; *p* < 0.001) and dAIH (male = 0.86 ± 0.17; female = 1.33 ± 0.22; *p* = 0.004). PKA regulatory subunit expression was also elevated in sham-exposed middle-aged females (male = 1.12 ± 0.29; female = 1.50 ± 0.37; *p* = 0.010). In contrast PKA catalytic subunit expression was higher in middle-aged females exposed to sham (male = 0.85 ± 0.22; female = 1.33 ± 0.31; p < 0.001) and dAIH (male = 0.80 ± 0.19; female = 1.13 ± 0.24; *p* = 0.006; Table 4).

**Fig. 6.**
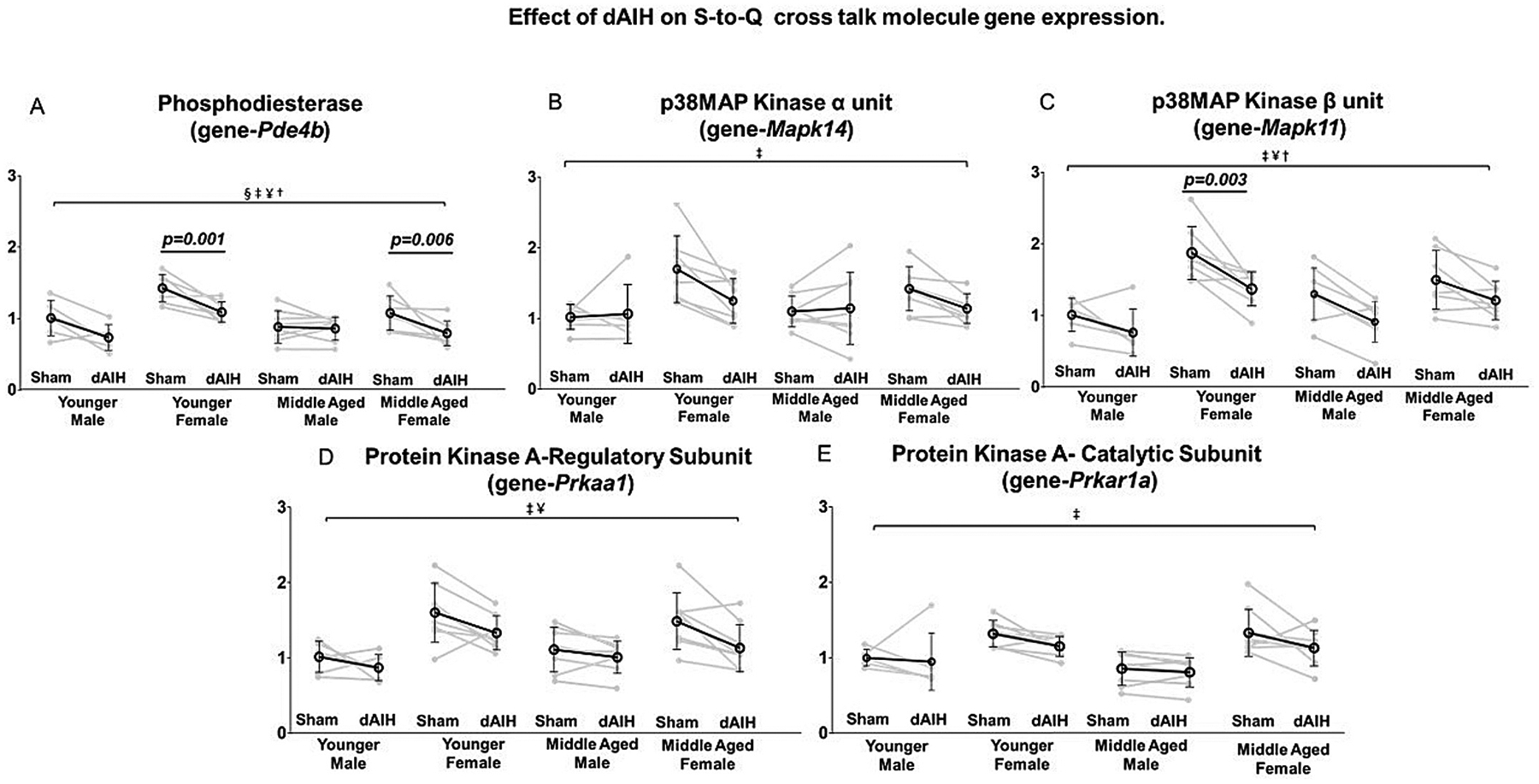
Effect of age, sex and daily AIH (dAIH) on genes associated with S to Q cross talk: phosphodiestease (*Pde4b*), p38Map kinase α (*Mapk14*), p38Map kinase β (*Mapk11*), PKA regulatory subunit (*Pkraa1*), and PKA catalytic subunit (*Prkar1a*). A significant main effect of age, sex, and treatment was observed in phosphodiesterase expression, with a significant age and sex interaction. dAIH treatment reduced phosphodiesterase expression in younger and middle aged females (Panel A). A significant main effect of sex was observed in p38Map kinase α expression (Panel B). A significant main effect of sex and treatment was observed in p38Map kinase β expression (Panel C). p38Map kinase β expression was significantly reduced with dAIH treatment in younger females (Panel C). A significant main effect of sex and treatment was observed in PKA regulatory subunit expression (Panel D) and PKA catalytic subunit expression. Additionally, a significant age-sex and age-sex-dAIH treatment interaction was observed with PKA catalytic subunit expression (Panel E). However, dAIH treatment had no significant effect on PKA regulatory or catalytic subunit in any individual group. §- Significant main effect of age; ‡ − Significant main effect of sex; ¥ - Significant main effect of treatment; † − Significant age*sex interaction; ʎ - Significant age*sex*treatment interaction.

An overall main effect of dAIH was detected in mRNA for phosphodiesterase-4b (MD = −0.23, F = 20.15, *p* < 0.001), p38MAPkinase β subunit (MD = −0.35, F = 18.32, *p* < 0.001), and PKA regulatory subunit (MD = −0.22, F = 8.24, p = 0.006; Table 2). The dAIH effects were primary observed as a significant reduction of phosphodiesterase-4b mRNA expression in both younger (Sham = 1.42 ± 0.19; dAIH = 0.94 ± 0.14; *p* = 0.001) and middle aged females (Sham = 1.07 ± 0.24; dAIH = 0.78 ± 0.17; p = 0.006) (Fig. 6A). Similarly, the dAIH effect on p38MAPkinase β subunit mRNA was primarily associated with a reduced expression in younger females (Sham = 1.68 ± 0.45; dAIH = 1.24 ± 0.31; *p* = 0.003) (Fig. 6C, Table 5).

### 3.4. Effect of age, sex and daily AIH (dAIH) on neuron-microglia signaling molecule: Fractalkine (Cx3cl1)

No main effect of age or sex was observed on fractalkine expression. Significant interaction effects of age × sex were detected in mRNA for fractalkine (β = −1.39, F = 10.67, *p* = 0.002), with greater expression in females. Pairwise comparisons demonstrate elevated fractalkine levels in dAIH-exposed middle-aged males (young = 0.63 ± 0.20; middle-aged = 1.52 ± 0.21; *p* = 0.001; Table 3). In young females, dAIH exposure increased fractalkine levels vs males (male = 0.63 ± 0.20; female = 1.38 ± 0.80; p = 0.004); however, in middle-aged females, dAIH was associated with lower fractalkine levels vs males (male = 1.52 ± 0.21; female = 0.85 ± 0.18; p = 0.005; Table 4).

**Fig. 7.**
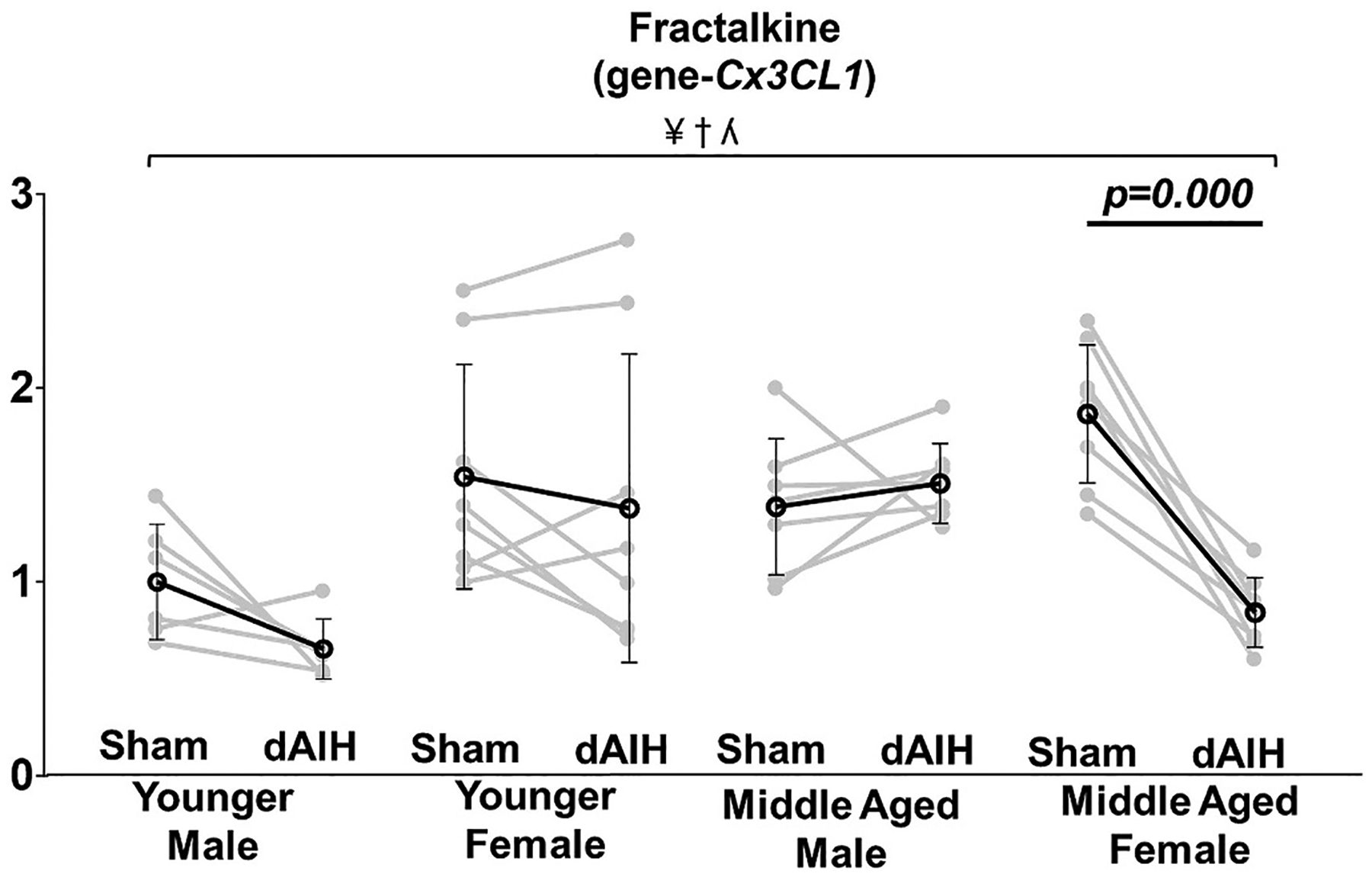
Effect of age, sex and daily AIH (dAIH) on neuron-microglia signaling molecule: Fractalkine (*Cx3cl1)*. A significant main effect of dAIH treatment was observed in fractalkine expression, with a significant age-sex and age-sex-dAIH treatment interaction. dAIH treatment significantly reduced fractalkine expression in middle-aged females. ¥ - Significant main effect of treatment; † − Significant age*sex interaction; ʎ - Significant age*sex*treatment interaction.

A significant interaction effect for age × sex × dAIH was observed in fractalkine mRNA (β = 1.26, F = 7.22, p = 0.010), along with a significant main effect of dAIH on fractalkine mRNA (MD = −0.39, F = 11.12, p = 0.002), which was due to a significant reduction in its expression in middle-aged females (Sham = 1.87 ± 0.35; dAIH = 0.85 ± 0.18; *p* < 0.000). Fractalkine mRNA expression did not change significantly in any other studied age and sex group (See Fig. 7, Table 5).

### 3.5. Age, sex and daily AIH (dAIH) effects on the pro-plasticity molecule

Overall no effect of age on BDNF and EPAC mRNA levels was observed. However, a significant effect of sex was found on BDNF (MD = 0.60, F = 16.23, p < 0.001) and EPAC (MD = 0.57, F = 36.59, p < 0.001) mRNA expression. BDNF mRNA was elevated in dAIH exposed younger females compared to age matched males (male = 0.70 ± 0.39; female = 1.27 ± 0.40; p < 0.001; Table 4). The age × sex interaction was significant for EPAC (β = −0.85, F = 11.56, p = 0.001)(Table 2).

**Fig. 8.**
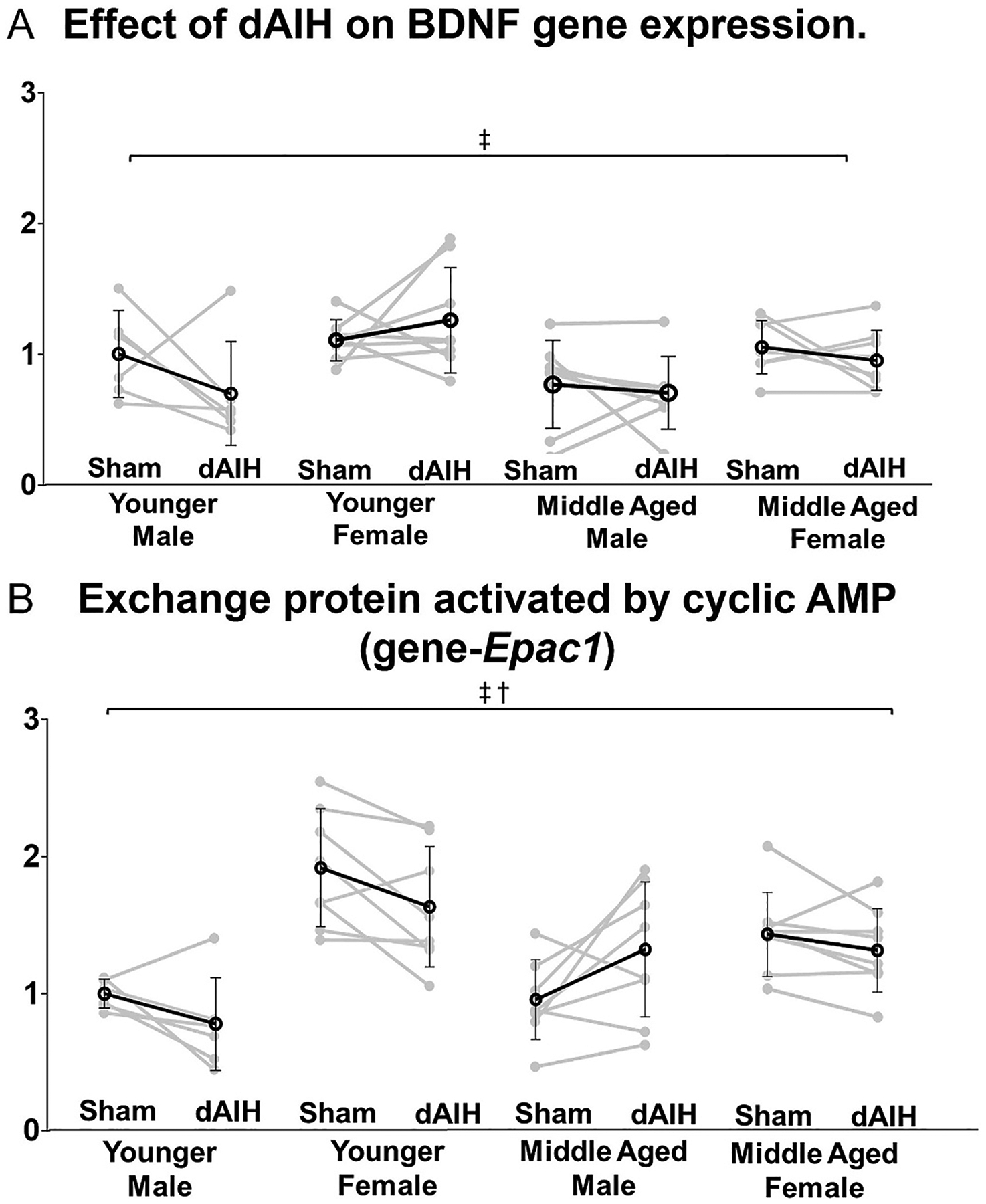
Age, sex and daily AIH (dAIH) effects on the pro-plasticity molecule, Brain derived neurotrophic factor (*Bdnf*) and Exchange protein activated by cyclic AMP (*Epac1*). A significant main effect of sex was observed in BDNF expression. dAIH treatment had no effect on BDNF mRNA expression in any of the tested groups (Panel A). A significant main effect of sex and an age-sex interaction were observed with EPAC (S-pathway driver activated by cAMP, Panel B). No other interaction effects were significant for BDNF and EPAC. §- Significant effect of age; ‡ − Significant effect of sex; † − Significant age*sex interaction.

In pairwise comparisons, EPAC expression was elevated in sham-exposed young females (young = 1.93 ± 0.43; middle-aged = 1.44 ± 0.31; p = 0.010) and dAIH-exposed middle-aged males (young = 0.78 ± 0.34; middle-aged = 1.31 ± 0.49; *p* = 0.009; Table 3). Sham-exposed young females had higher EPAC levels vs males (male = 1.00 ± 0.11; female = 1.93 ± 0.43; p < 0.001), as did sham-exposed middle-aged females (male = 0.95 ± 0.29; female = 1.44 ± 0.31; p = 0.009). Young dAIH-exposed females also had elevated EPAC mRNA vs males (male = 0.78 ± 0.34; female = 1.64 ± 0.44; p < 0.001; Table 4). However, no dAIH effects on BDNF (Fig. 8A) or EPAC mRNA (Fig. 8B) were detected within any age or sex cohort (Table 5).

### 3.6. Correlation of serum estradiol with Q-S cross-talk molecules

**Fig. 9.**
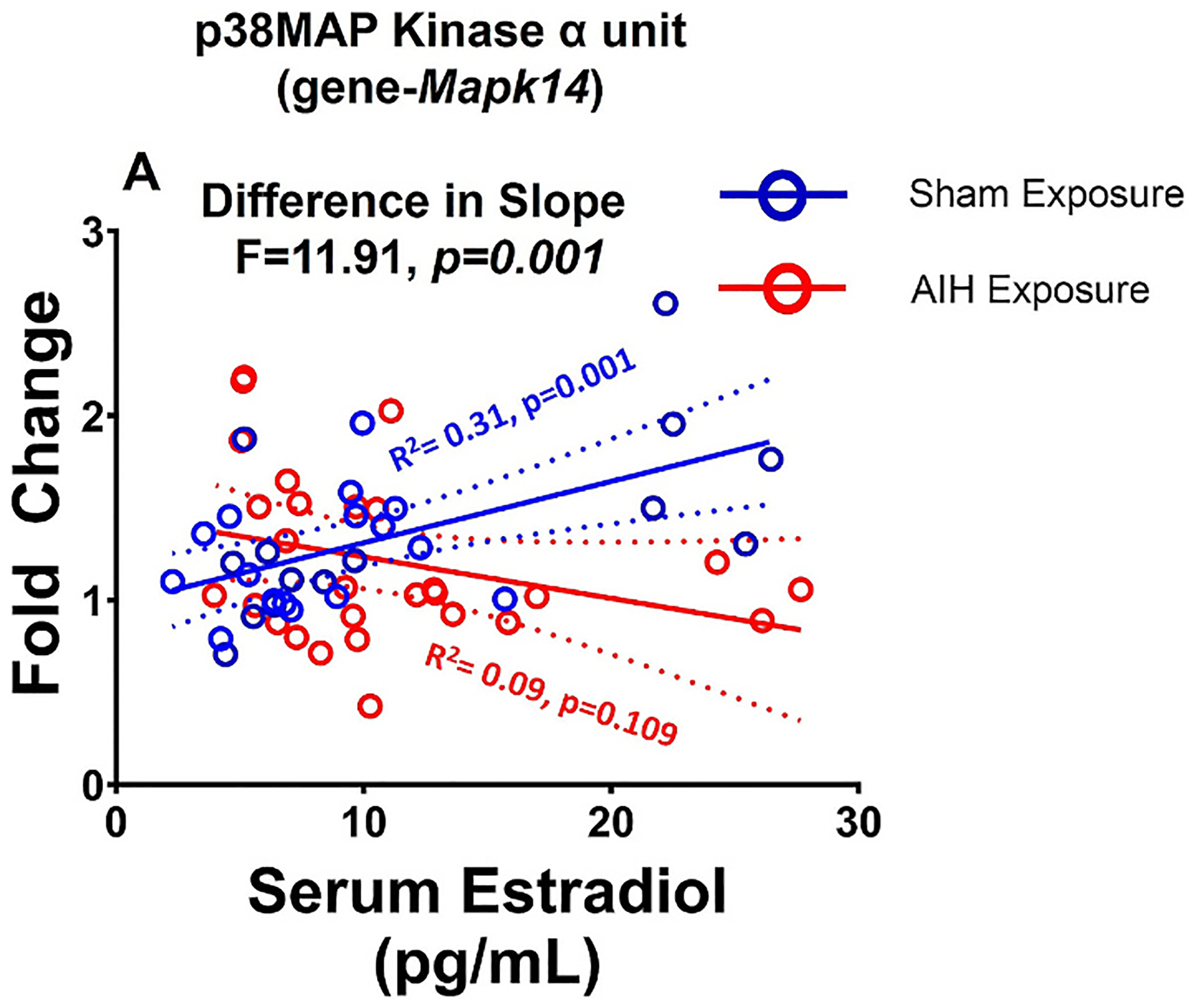
Correlation of serum estradiol with dAIH induced changes in p38 MAP kinase α unit (*Mapk14*) expression. A significant positive correlation was observed between p38 MAP kinase α and serum estradiol in sham exposed rats. In rats exposed to dAIH, estradiol level negatively correlated with p38 MAP kinase α expression.

In rats not exposed to dAIH, serum estradiol positively correlated with EPAC (R^2^ = 0.29, p = 0.002) and p38MAPkinase α subunit (R^2^ = 0.31, *p* = 0.000; Fig. 9, Table 6).

### 3.7. Correlation of serum estradiol with dAIH effects

A significant correlation was observed between serum estradiol and the dAIH-induced decrease in the p38MAPkinase α subunit (Sham: R^2^ = 0.31; dAIH: R^2^ = 0.09, slope difference F = 11.91, p = 0.001; Fig. 9). However, serum estradiol was not correlated with dAIH-induced changes in Q- or S-pathway receptors (5HT2A, 5HT2B, 5HT7, and A2A), Q to S cross-talk molecules (PKCδ, NADPH-p47phox, NADPH-gp91phox), S to Q cross-talk molecules (EPAC, PKA Reg, PKA Cat subunit), phosphodiesterase-4b, fractalkine ligand, or BDNF (refer to Table 6).

## 4. Discussion

### 4.1. Changes in Q pathway ligand/receptor and Q to S plasticity-regulating molecules with age and sex

Our results show that BDNF mRNA was only elevated in dAIH-exposed young females compared to age-matched males. Prior work demonstrate elevated BDNF levels in hippocampus, ventromedial hypothalamus, cortex and amygdala in female vs male rats (Bakos et al., 2009; Bland et al., 2005; Liu et al., 2014; Snigdha et al., 2011). The implications of elevated BDNF in young female appears to be counter-intuitive given that young females have a lower capacity to express pLTF compared to age-matched males. We speculate that lower pLTF in young females arise from upregulation of multiple S to Q crosstalk inhibition as discussed in later sections.

Daily AIH increased 5HT2B expression in middle aged males, but not in other groups. NADPH oxidase (NOX: p47phox) was elevated only in middle aged males, with or without dAIH. Increased 5HT2B receptor-expression may increase the capacity for Q pathway depended pLTF via NOX-linked reactive oxygen species (ROS) formation (MacFarlane et al., 2011), increased NOX activity and ROS formation could also inhibit adenyl cyclase activity and cAMP formation, thereby undermining the S-pathway (Perim et al., 2018; Haenen et al., 1990). Since p47phox (NOX-associated mRNA) was significantly higher in middle aged males, a sex specific mechanism of NOX-mediated Q to S cross-talk inhibition cannot be ruled out.

Since PKCδ constrains 5HT7 receptor-induced S-pathway to phrenic motor facilitation (Perim et al., 2019), observed PKCδ elevations in middle-aged males (regardless of dAIH exposure) and dAIH exposed young females, suggest the possibility that dAIH favors Q to S cross-talk inhibition in middle aged males vs young females. PKCδ has a catalytic domain and a highly reactive regulatory domain that reacts with diacylglycerols to inhibit adenyl cyclase activity and cAMP formation (Lai et al., 1997; Shanmugasundararaj et al., 2012). PKCδ is also a downstream target of estrogen receptors that regulates mitochondrial respiration by controlling ROS formation (Evola et al., 2018; Jeong and Shin, 2012; Rex et al., 2010). Although PKC expression is typically thought to decrease with age due to epigenetic modifications, the PKCδ isoform is an exception (Pascale et al., 1996; Patterson et al., 2010). Indeed, our results are consistent with a prior report demonstrating that PKCδ is unaffected by age (Pascale et al., 1996). Collectively, our data suggest Q pathway dominance and greater Q to S cross-talk inhibition in young females and middle age males.

### 4.2. Changes in S pathway ligand/receptor and S to Q plasticity-regulating molecules with age and sex

Spinal A2A receptor inhibition doubles moderate AIH-induced phrenic LTF in young (Hoffman et al., 2010) and its effect is even greater in old male rats (Marciante and Mitchell, 2023a). Our finding that A2A receptor mRNA is higher in young females, but increases with age in males is consistent with these functional observations. Combined with reports of increased extracellular adenosine concentration with age in male rats (Castillo et al., 2009; Sebastião et al., 2000; Stockwell et al., 2017; Marciante and Mitchell, 2023a), greater S pathway activation may substantially constrain the Q-pathway to pLTF in older rats (Marciante and Mitchell, 2023a). Our observation that dAIH reduced A2A receptor expression in middle aged females could help explain why mild chronic intermittent hypoxia restores moderate AIH-induced pLTF in geriatric females (Zabka et al., 2003).

p38 MAP kinase, a downstream signaling molecule from A2A receptors (Kralova et al., 2008; Seven et al., 2020) can cross-talk inhibit Q pathway-dependent pLTF (Huxtable et al., 2015; Burrowes and Mitchell, unpublished). Thus, higher p38MAP kinase α and β subunit expression in young females suggests the potential for greater p38MAP kinase-dependent S to Q cross-talk inhibition, suppressing mAIH-induced pLTF in females. Interestingly, p38MAP kinase β subunit expression was highest in young females and significantly reduced with age and dAIH exposure. Differential p38MAP kinase β subunit expression could contribute to increased moderate AIH-induced pLTF in middle-aged females (Behan et al., 2002; Behan et al., 2003; Dougherty et al., 2017; Zabka et al., 2001a, 2001b; Zabka et al., 2006).

During moderate hypoxia, increased cAMP activates PKA, an important S to Q cross-talk molecule (Fields and Mitchell, 2017; Hoffman and Mitchell, 2013). Increased cAMP binds to PKA regulatory subunits (PKA Reg), inducing PKA conformational changes that free the catalytic subunit (PKA Cat) to engage in enzymatic activity (Shaikh et al., 2012). Once activated, PKA Cat phosphorylates cytosolic p47phox subunit (Kandel, 2012), inhibiting the NOX complex and ROS formation (Bengis-Garber and Gruener, 1996; Kim et al., 2007; Nogueira-Machado, 2003).

During severe hypoxia substantial increases in intracellular cAMP activate EPAC, a necessary step in driving the S pathway to phrenic motor facilitation (Fields and Mitchell, 2017; Fields et al., 2015). This pathway to phrenic motor facilitation is mechanistically distinct from serotonin-induced PKA-ERK-BDNF signaling pathway (Hoffman and Mitchell, 2011). Although there were no changes in PKA Reg or PKA Cat mRNA with age, greater EPAC expression with age in young females and dAIH-exposed middle-aged males suggest greater capacity for S-pathway driven phrenic motor plasticity and, possibly, increased S to Q cross-talk inhibition in young females.

Since PKA activity is inhibited by the long isoform of phosphodiesterase-4b (Fertig and Baillie, 2018), phosphodiesterase-4b likely plays an important role in minimizing PKA mediated S to Q cross-talk interactions. Thus, decreased phosphodiesterase-4 mRNA with age in females may reflect increased PKA-dependent S to Q cross-talk inhibition. Conversely, greater phosphodiesterase-4 in younger females suggests the possibility of compensatory mechanisms that offset PKA-mediated S to Q cross-talk inhibition. Since dAIH exposure downregulated phosphodiesterase-4 mRNA in young and middle-aged females, greater S to Q mediated pLTF is possible in this cohort. The overall impact of gene expression changes on AIH-induced plasticity will likely depend on complex dynamic interactions between age and sex.

Recent studies indicate that phrenic motor neurons release fractalkine (*Cx3cl1*), a chemokine uniquely expressed in neurons (Marciante et al., 2024). Fractalkine activates microglial fractalkine receptors and triggers microglia-dependent adenosine accumulation necessary for S to Q cross-talk inhibition with moderate AIH, and AIH-induced pLTF with severe AIH (i.e., S-pathway). Thus, higher fractalkine expression suggests greater suppression of Q-pathway-driven pLTF in middle-aged males and dAIH exposed young females.

Taken together, young females appear to have greater S to Q cross talk inhibition as suggested by their elevated levels of A2A receptor, p38Mapkinase β subunit, PKA Reg, PKA Cat, EPAC and fractalkine mRNA. However, the potential impact of these differences will depend on the specific cells giving rise to observed changes in the homogenates studied here. Powerful cross talk inhibition may override proplasticity effects arising from higher BDNF levels in young females. Thus, despite potentially greater capacity for Q pathway driven plasticity may be subject to greater regulation (i.e., increased cross-talk inhibition). With advancing age declines in these same S to Q cross talk molecules may favor pLTF, as reported previously in middle aged females (Behan et al., 2002; Behan et al., 2003; Dougherty et al., 2017; Zabka et al., 2001a, 2001b; Zabka et al., 2006).

### 4.3. dAIH effects on pLTF metaplasticity

**Fig. 10.**
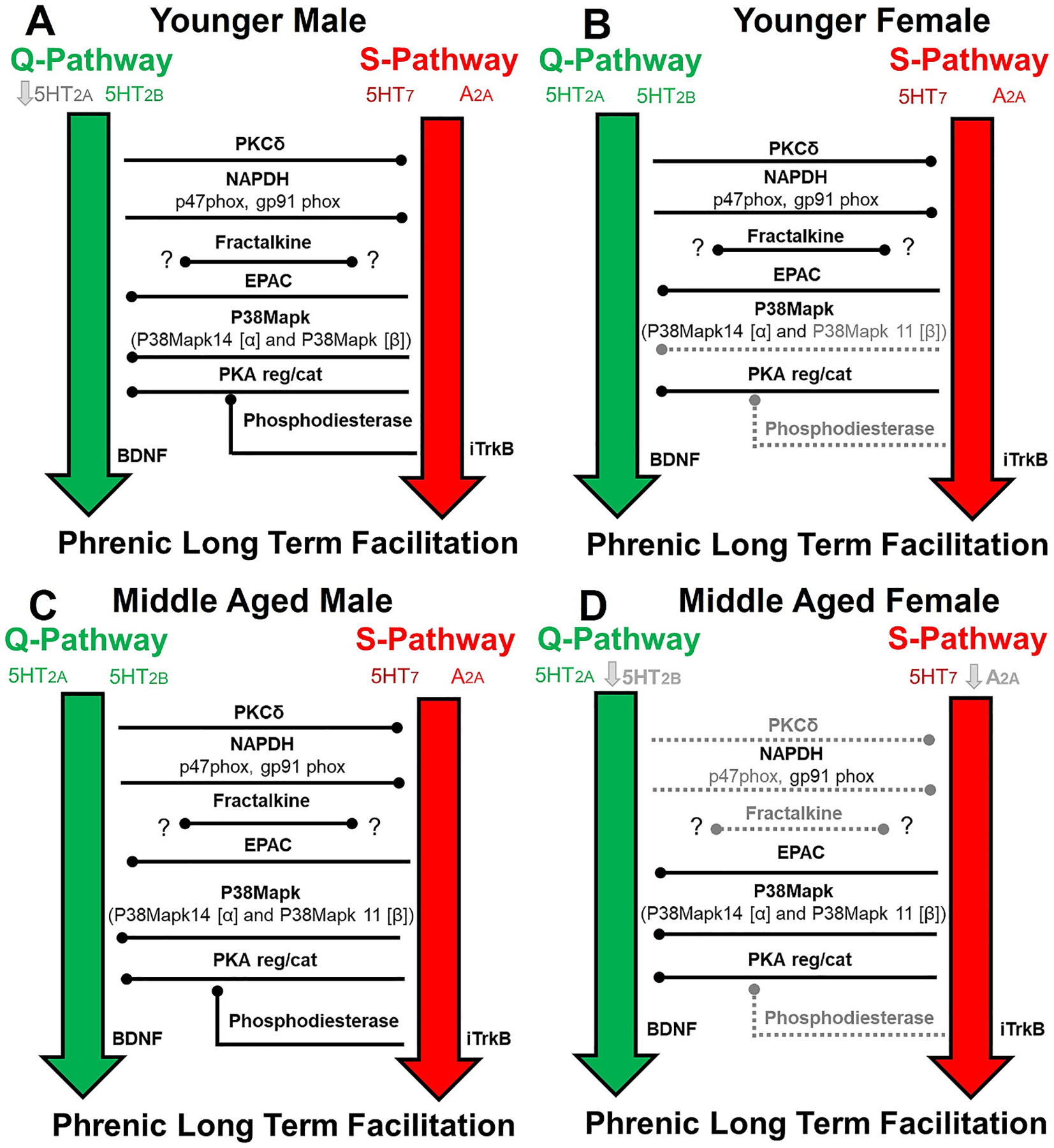
Conceptual diagram of dAIH effects on molecules engaged in Q-S cross talk inhibition in young (3 month) and middle aged (12 months) male and female rats. In young males, dAIH exposure significantly reduced Q pathway associated 5HT2A receptor expression (Panel A). In young females, dAIH significantly reduced S to Q cross talk molecules – p38Map kinase β (*Mapk11*) and phosphodiesterase (*Pde4b*) expression. Reduced phosphodiesterase expression could indirectly increase PKA or EPAC activity which could in turn increase S to Q inhibition (Panel B). In middle aged males, no significant effect of dAIH exposure was observed (Panel C). In contrast widespread gene expression changes with dAIH exposure were observed in middle aged females. dAIH reduced expression of 5HT2B and A2A receptor, Q to S cross talk molecules–PKCδ (*Prkcd*) and NADPH-p47 (*Ncf1*), S to Q cross-talk molecules phosphodiesterase 4b, and fractalkine (*Cx3cl1*) (Panel D).

A conceptual diagram of dAIH effects on molecules engaged in the Q & S pathways, as well as Q to S and S to Q cross talk inhibition, in young (3 month) and middle aged (12 months) male and female rats is summarized in Fig. 10. Metaplasticity induced by dAIH pretreatment has been primarily studied in young adult male rats (Hoffman et al., 2010; MacFarlane et al., 2018; Mitchell and Baker, 2022; Satriotomo et al., 2012). Information concerning Q or S-pathway molecules in middle-age or geriatric males or females are scarce (but see: Marciante et al., 2023; Marciante and Mitchell, 2023a). Multiple single session AIH studies suggest profound age-dependent sexual dimorphism in pLTF expression. For instance, moderate AIH-induced pLTF decreases between 3 (young adult) and 12 months (middle age) in males, but increases with age in female rats (Behan et al., 2002; Behan et al., 2003; Dougherty et al., 2017; Zabka et al., 2001a, 2001b; Zabka et al., 2006). Since pLTF also varies with estrus cycle (Zabka et al., 2001b; Dougherty et al., 2017), the impact of age, sex and estrus cycle on dAIH-induced metaplasticity must be considered.

### 4.4. dAIH-induced changes in mRNAs for Q pathway and Q to S cross-talk molecules

A dAIH associated downregulation of Q pathway-associated 5HT2A receptors (Tadjalli and Mitchell, 2019b) in younger males, the model most widely used to study AIH-induced meta-plasticity in rodents. In contrast, a downregulation of S pathway-associated A2A receptors (Golder et al., 2008) in middle aged females with dAIH (a major source of cross-talk inhibition in younger males, Fields and Mitchell, 2015) suggests complex effects on a primary driver of mAIH-induced plasticity. Lack of dAIH effects on 5HT2B, 5HT7 and BDNF mRNA suggest that these elements driving (on constraining) plasticity in single session AIH are still likely involved after repeated AIH exposure. The lack of increase in BDNF mRNA may seem at variance from prior reports that BDNF protein (assessed via immunohistochemical or ELISA assays) increases following dAIH (Lovett-Barr et al., 2012; Wilkerson and Mitchell, 2009). Differences between mRNA and protein expression are not uncommon, and could reflect temporal dissociation between mRNA and protein, or that BDNF is increased via translational versus transcriptional regulation with dAIH (Baker-Herman and Mitchell, 2002; Tadjalli and Mitchell, 2019a). On the other hand, our finding supports a prior report that BDNF and 5HT7 mRNA in ventral cervical homogenates is unaffected by repetitive AIH preconditioning (3× per week for 4 weeks) in male rats (MacFarlane et al., 2018).

Downregulation in spinal A2A receptors in middle aged females (Fig. 4) could help explain an important mechanism whereby middle aged females enhance pLTF following repetitive intermittent hypoxia (5 min episodes, 12 h per day in the active phase; Zabka et al., 2001b).

### 4.5. dAIH effects on Q to S cross-talk molecules

dAIH pretreatment downregulation of Q to S cross-talk molecules including PKCδ and NADPH-associated p47 expression were significant only in middle-aged females, consistent with enhanced AIH-induced pLTF in middle-age females (Zabka et al., 2001b).

### 4.6. dAIH effects on S to Q cross-talk molecules

dAIH effects on S-Q cross-talk were largely influenced by the age and biological sex; fractalkine expression was only reduced in middle aged females, confirming the observation that AIH induced plasticity is enhanced in middle-age females (Zabka et al., 2001a, 2001b). Decreased p38MAP kinase β subunit expression in younger females indicates multiple dAIH effects across age, particularly in females. Decreased p38MAP kinase mRNA suggests the possibility of decreased neuroinflammation and, thus, Q-pathway inhibition (Agosto-Marlin et al., 2018). On the other hand, dAIH effects in younger females could be constrained by decreased phosphodiesterase-4b expression, possibly increasing PKA activation and S to Q cross-talk inhibition (Zabka et al., 2001b). Decreased phosphodiesterase-4b may also increase cAMP, sufficiently, to activate EPAC (which has a lower cAMP affinity than PKA, Smith et al., 2005). Activated EPAC thereby can trigger the S-pathway to phrenic motor facilitation, which is consistent with the apparent S-pathway contribution to enhanced pLTF in rats exposed to repetitive AIH (3× per week, 4 weeks; (MacFarlane et al., 2018; Perim et al., 2020), or daily AIH for 14 days (Perim et al., 2021b). The functional impact of dAIH-reduced phosphodiesterase-4b is of considerable interest, but awaits further experimentation. The role of PKA Reg subunit in regulating pLTF across age and sex cohorts remains unclear since there were no significant decreases in PKA Reg subunit mRNA in any dAIH-exposed group.

### 4.7. Correlation of serum estradiol with dAIH-altered gene expression

A growing body of evidence demonstrates female sex hormone effects on AIH-induced phrenic motor plasticity (Behan et al., 2002; Lurk et al., 2022; McIntosh and Dougherty, 2019). In females, AIH induced ventilatory facilitation is detected only during proestrus, when systemic estrogen levels are high (Dougherty et al., 2017; McIntosh and Dougherty, 2019). Similarly, pLTF in middle-aged females (Zabka et al., 2001b) requires a surge in systemic estradiol as rats transition to reproductive senescence (Frick, 2009). In males, gonadectomy abolishes pLTF in young adult males, an effect restored by supplemental testosterone; since restoration of pLTF requires CNS conversion of testosterone to estrogen via aromatase activity (Zabka et al., 2006), CNS estrogen likely impacts key molecules regulating the Q and/or S-pathways and their cross-talk inhibition independently from circulating estrogen levels.

We did not study estrous cycle effects in the present study, but correlated dAIH-induced mRNA changes with serum estradiol levels. Interestingly, we observed that animals exposed to dAIH were disproportionately in the proestrus phase compared to sham exposed animals, indicting that dAIH exposure may influence estrus cycling. In naïve rats, serum estradiol positively correlated with EPAC and p38MAP kinase α subunit mRNA expression. A significant correlation between serum estradiol and dAIH-induced changes was observed only for p38MAP kinase α subunit. Decreased p38MAPkinase α subunit after dAIH suggests pro-plasticity and neuroprotective dAIH effects may be under the influence of circulating estradiol. A controlled experiment with carefully tittered serum estradiol concentration is needed to confirm these observations.

## 5. Limitations

We did not perform experiments that directly test dAIH effects on AIH-induced respiratory motor plasticity in the present study since we did not wish to confound results with the possible effects of prolonged anesthesia (Aravindan et al., 2006), hyperoxia (FiO2 = 0.5) (Wright and Dennery, 2009), blood loss (Shenkar and Abraham, 1993), or vagotomy (O’Leary et al., 2018). Thus, we cannot correlate ventral cervical spinal cord mRNA with direct measures of neuroplasticity in the same animals. Instead, we carefully analyzed differences in molecular underpinnings of plasticity in the ventral cervical (C3-C6) spinal cord in rats exposed to 14 days of daily Sham or AIH, and discussed those changes in relation to prior literature accounts of AIH-induced pLTF across ages, sex and dAIH pre-treatment.

Since ventral cervical homogenates contain multiple cell types, including neurons, microglia, astrocytes, oligodendrocytes and other cells, tissue homogenates reflect the molecular environment near phrenic motoneurons versus changes in phrenic motor neuron expression per se. Thus, while mRNA changes reported here may be linked to phrenic motoneuron plasticity, mRNA changes may in fact reflect expression in other cell types. For example, we recently demonstrated that microglia per say are critical in the adenosine response (and activation of the S pathway) with intermittent hypoxia, largely due to reciprocal fractalkine/adenosine signaling (Marciante et al., 2024). Further, estradiol in the ventral cervical spinal cord may differ from serum levels. Although progesterone regulates ventilation (Behan et al., 2003), the role of progesterone in AIH induced respiratory motor plasticity is difficult to study due to truncated duration of the estrus cycle in rats (~4 days) vs the human menstrual cycle (~28 days) (McIntosh and Dougherty, 2019). Gonadectomy-induced transcriptome changes in the ventral cervical spinal cord of male and female rats were recently published (Miller et al., 2022). However, instead of comprehensive gene expression analytics such as RNAseq or QuantSeq, we focused on specific mRNAs from known pro-plasticity molecules in the ventral cervical cord, which harbors the phrenic motor nucleus. Other genes (Miller et al., 2022)), and molecules not included in this analysis, could be impacted by dAIH, age, and/or sex. In the end, for any mRNA to impact plasticity, it must be translated into active proteins. Thus, follow-up neurophysiological studies are needed to determine if the translated proteins from these mRNAs underlie pLTF metaplasticity induced by dAIH.

## 6. Conclusion

Based on this extensive study of age–dependent sexual dimorphism in daily AIH responses of molecules known to regulate phrenic motor plasticity, we conclude that daily AIH downregulates Q-S cross-talk molecules in an age and sex dependent manner, with the greatest downregulation in middle-aged females—when plasticity is at its peak (Zabka et al., 2001b). Based on correlations of estradiol with dAIH effects, we speculate that these variables regulate AIH-induced pLTF at least in part by suppressing p38MAP kinase activity. This study begins to address an important question, but leaves open other important questions such as: 1) is there a causal relationship between estradiol and the pro-plasticity molecules? 2) What cell types are changing expression in response to daily AIH and/or age/sex; and 3) how do changes in these molecules impact plasticity? The novel findings presented here (and answers to the above questions) will advance our understanding of pLTF, and inform translational research concerning the therapeutic potential of daily AIH to treat individuals of different ages and sex to improve breathing ability.

## Figures and Tables

**Table 1:** TaqMan oligonucleotide primers and probe sets used for PCR.

Protein encoded	Gene Symbol	Role	TaqMan Assay ID	Amplicon length
5HT2A	*Htr2a*	Q pathway receptor	Rn00568473_m1	71
5HT2B	*Htr2b*	Q pathway receptor	Rn00691836_m1	81
5HT7	*Htr7*	S pathway receptor	Rn00576048_m1	85
Adenosine 2A	*adora2a*	S pathway receptor	Rn00583935_m1	58
Protein kinase C δ	*Prkcd*	Q to S crosstalk	Rn00440891_m1	58
NADPH-p47phox	*ncf1*	Q to S crosstalk	Rn00586945_m1	105
NADPH-gp91phox	*Cybb*	Q to S crosstalk	Rn00576710_m1	77
phosphodiesterase 4B	*pde4b*	S to Q crosstalk	Rn00566785_m1	70
p38MapKinase α subunit	*Mapk14*	S to Q crosstalk	Rn00578842_m1	87
p38MapKinase β subunit	*Mapk11*	S to Q crosstalk	Rn01407663_g1	65
PKA Regulatory Subunit	*Prkaa1*	S to Q crosstalk	Rn00566036_m1	84
PKA Catalytic Subunit	*Prkar1a*	S to Q crosstalk	Rn01402558_m1	69
Fractalkine	*Cx3cl1*	S to Q crosstalk	Rn00593186_m1	74
BDNF	*Bdnf*	Plasticity Molecule	Rn02531967_s1	142
Exchange protein directly activated by cAMP	*Epac1*	S to Q crosstalk	Rn00572463_m1	65
Eukaryotic Ribosomal RNA	*18 s*	Reference gene	Hs99999901_s1	187

**Table 2:** Effect of age-sex-dAIH treatment interaction on pro-plasticity gene expression. Significance p < 0.05 (Bonferroni Correction)

				Main effect									Interaction effect								
	Age			Sex			dAIH			Age*Sex			Age-dAIH			Sex*dAIH			Age*Sex*dAIH		
Protein Encoded *(gene)*	F	Parameter Estimates	*Sig*.	F	Parameter Estimates	*Sig*.	F	Parameter Estimates	*Sig*.	F	Parameter Estimates	*Sig*.	F	Parameter Estimates	*Sig*.	F	Parameter Estimates	*Sig*.	F	Parameter Estimates	*Sig*.
5HT2A (*Htr2a*)	0.36	0.12	*0.551*	3.86	−0.16	*0.055*	11.06	0.24	***0.002***	0.42	−0.12	*0.522*	0.16	−0.25	*0.688*	2.135	−0.14	*0.15*	2.48	0.4	*0.121*
5HT2B (*Htr2b*)	24.81	−0.06	<***0.001***	0.14	0.32	*0.708*	7.48	0.57	***0.009***	4.5	−0.78	*0.039*	1.29	−0.55	*0.262*	0.033	−0.24	*0.856*	1.48	0.56	*0.23*
5HT7 (*HtrT*)	0.37	0.03	*0.548*	2.46	0.2	*0.123*	3.97	0.29	*0.052*	0.77	0.08	*0.385*	1.53	−0.01	*0.223*	0.25	0.161	*0.619*	1.46	−0.55	*0.233*
A2A (*Adora2a*)	10.19	0.19	***0.002***	52.04	−0.06	<***0.001***	10.04	0.65	***0.003***	6.66	−0.79	*0.013*	0.55	−0.48	*0.463*	3.049	−0.649	*0.087*	4.55	0.71	*0.038*
PKCC δ (*Pkcd*)	4.92	0.74	0.031	8.09	0.45	***0.006***	14.74	1.05	<***0.001***	13.73	−1.4	<***0.001***	6.52	−1.15	*0.014*	0.275	−0.707	*0.602*	7.46	1.19	***0.009***
p47phox (*Ncfl*)	35.72	−0.32	<***0.001***	7.19	−0.01	***0.01***	15.04	0.63	<***0.001***	1.47	−0.31	*0.231*	1.4	−0.3	*0.242*	1.013	−0.269	*0.319*	0.14	0.14	*0.715*
gp91phox(*Cybb*)	17.5	−0.44	<***0.001***	4.35	−0.18	*0.042*	6.13	0.26	*0.017*	0.46	0.08	*0.5*	0	0.08	*0.989*	0.019	0.45	*0.891*	0.11	−0.15	*0.739*
phosphodi esterase 4B (*Pde46*)	9.45	0.3	***0.003***	18.36	0.83	<***0.001***	20.15	0.28	<***0.001***	11.32	−0.44	***0.001***	2.21	0.05	*0.143*	2.519	−0.259	*0.119*	0.92	0.2	*0.342*
p38MapKinase β subunit (*Mapk11*)	0.03	0.15	*0.855*	34.98	−0.3	<***0.001***	18.32	0.29	<***0.001***	8.44	−0.31	***0.005***	0.05	0.22	*0.82*	0.206	0.102	*0.652*	1.14	−0.36	*0.292*
p38MapKinase α subunit (*Mapk14*)	0.34	0.12	*0.565*	10.18	0.02	***0.002***	3.05	0.28	*0.087*	2.66	−0.21	*0.109*	0.2	0.17	*0.653*	5.01	−0.325	0.03	0.21	−0.17	*0.651*
PKA Regulatory Subunit (*Prkaa1*)	0.00	−0.13	*0.993*	27.63	−0.132	<***0.001***	8.28	0.35	***0.006***	3.34	−0.34	*0.073*	0.02	−0.08	*0.892*	1.576	−0.252	*0.215*	0.18	0.13	*0.673*
PKA Catalytic Subunit (*Prkar1a*)	1.48	0.1	*0.231*	30.32	−0.33	<***0.001***	3.77	0.2	*0.058*	1.81	0.14	*0.184*	0.02	−0.04	*0.889*	1.32	−0.156	*0.256*	0.03	0.04	*0.876*
Fractalkine (*Cx3c11*)	4.97	0.57	0.03	4.64	0.67	*0.036*	11.12	1.06	***0.002***	10.67	−1.39	***0.002***	1.32	−0.9	*0.256*	3.62	−1.072	*0.063*	7.22	1.26	***0.01***
BDNF (*Bdnf*)	3.28	0.19	0.076	16.23	−0.06	<***0.001***	1.01	0.65	*0.32*	0.19	−0.79	*0.668*	0	−0.48	*0.965*	1.828	−0.649	*0.182*	2.5	0.71	*0.12*
EPAC (*Epac1*)	0.76	0.32	*0.389*	36.59	−0.01	***0.000***	0.47	0.12	*0.498*	11.56	−0.85	***0.001***	3.97	0.17	*0.051*	2.074	−0.483	*0.156*	1.24	0.42	*0.271*

**Table 3:** Effect of age on pro-plasticity gene expression. Significance p ≤ 0.01 (Bonferroni Correction).

Protein Encoded (*gene*)	Male Sham			Female Sham			Male dAIH			Female dAIH		
	Young	Middle-Aged	*p*	Young	Middle-Aged	*p*	Young	Middle-Aged	*p*	Young	Middle-Aged	*p*
5HT2A (*Htr2a*)	1.00 ± 0.44	0.85 ± 0.26	*0.250*	0.89 ± 0.26	1.02 ± 0.23	*0.297*	0.63 ± 0.17	0.62 ± 0.19	*0.971*	0.90 ± 0.15	0.78 ± 0.14	*0.319*
5HT2B (*Htr2b*)	1.00 ± 0.30	1.82 ± 0.64	***0.001***	1.13 ± 0.22	1.73 ± 0.54	***0.009***	0.65 ± 0.31	1.49 ± 0.60	***0.001***	1.1 ± 0.36	1.16 ± 0.33	*0.792*
5HT7 (*Htr7*)	0.88 ± 0.61	1.45 ± 0.73	*0.068*	1.12 ± 0.45	1.09 ± 0.32	*0.898*	1.09 ± 0.76	1.00. ± 0.41	*0.239*	0.84 ± 0.42	0.80 ± 0.16	*0.876*
A2A (*Adora2a*)	1.00 ± 0.25	1.37 ± 0.39	*0.040*	1.79 ± 0.28	1.6 ± 0.38	*0.076*	0.76 ± 0.20	1.36 ± 0.39	***0.001***	1.62 ± 0.25	1.07 ± 0.33	*0.245*
PKCC δ (*Pkcd*)	1.00 ± 0.28	1.60 ± 0.69	***0.008***	1.47 ± 0.40	1.89 ± 0.35	*0.053*	0.62 ± 0.03	1.28 ± 0.13	***0.005***	1.57 ± 0.52	0.8 ± 0.17	**<*0.001***
p47phox (*Ncf1*)	1.00 ± 0.25	1.80 ± 0.59	**<*0.001***	1.44 ± 0.27	1.94 ± 0.55	*0.002*	0.80 ± 0.23	1.40 ± 0.44	***0.003***	1.12 ± 0.13	1.35 ± 0.34	*0.100*
gp91phox (*Cybb*)	1.00 ± 0.25	1.08 ± 0.47	*0.016*	1.2 ± 0.70	1.8 ± 0.66	*0.107*	0.77 ± 0.33	1.29 ± 0.51	*0.033*	1.01 ± 0.47	1.45 ± 0.44	*0.050*
phosphodiesterase 4B (*Pde4b*)	1.00 ± 0.23	1.07 ± 0.22	*0.303*	1.42 ± 0.19	1.07 ± 0.22	**<*0.001***	0.73 ± 0.16	0.87 ± 0.15	*0.193*	1.09 ± 0.14	0.78 ± 0.16	***0.003***
p38MapKinase β subunit (*Mapk11*)	1.08 ± 1.17	1.30 ± 0.36	*0.092*	1.86 ± 0.37	1.46 ± 0.40	*0.027*	0.66 ± 1.39	0.91 ± 0.28	*0.368*	1.36 ± 0.24	1.15 ± 0.32	*0.354*
p38MapKinase α subunit (*Mapk14*)	1.04 ± 1.24	1.10 ± 0.22	*0.613*	1.68 ± 0.45	1.38 ± 0.30	*0.111*	0.93 ± 1.24	1.14 ± 0.51	*0.616*	1.24 ± 0.31	1.07 ± 0.26	*0.501*
PKA Regulatory Subunit (*Prkaa1*)	1.00 ± 0.21	1.12 ± 0.29	*0.464*	1.60 ± 0.39	1.50 ± 0.37	*0.509*	0.86 ± 0.17	1.02 ± 0.07	*0.315*	1.33 ± 0.22	1.15 ± 0.11	*0.218*
PKA Catalytic Subunit (*Prkar1a*)	1.00 ± 0.11	0.85 ± 0.22	*0.220*	1.31 ± 0.17	1.33. ± 0.31	*0.824*	0.95 ± 0.37	0.80 ± 0.19	*0.226*	1.14 ± 0.13	1.13 ± 0.24	*0.931*
Fractalkine (*Cx3cl1*)	0.96 ± 0.53	1.40 ± 0.35	*0.058*	1.54 ± 0.58	1.87 ± 0.35	*0.159*	0.63 ± 0.20	1.52 ± 0.21	***0.001***	1.38 ± 0.80	0.85 ± 0.18	*0.016*
BDNF (*Bdnf*)	1.00 ± 0.33	0.77 ± 0.34	*0.166*	1.12 ± 0.16	1.07 ± 0.20	*0.750*	0.70 ± 0.39	0.71 ± 0.28	*0.940*	1.27 ± 0.40	0.97 ± 0.23	*0.049*
EPAC (*Epac1*)	1.00 ± 0.11	0.95 ± 0.29	*0.783*	1.925 ± 0.43	1.44 ± 0.31	***0.010***	0.78 ± 0.34	1.31 ± 0.49	***0.009***	1.64 ± 0.44	1.32 ± 0.11	*0.084*

**Table 4:** Effect of sex on pro-plasticity gene expression. Significance p ≤ 0.01 (Bonferroni Correction).

Protein Encoded (*gene*)	Young Sham			Young dAIH			Middle Age Sham			Middle Age dAIH		
	Male	Female	*p*	Male	Female	*p*	Male	Female	*p*	Male	Female	*p*
5HT2A (*Htr2a*)	1.00 ± 0.44	0.89 ± 0.26	*0.412*	0.63 ± 0.17	0.90 ± 0.15	*0.041*	0.85 ± 0.26	1.02 ± 0.23	*0.163*	0.62 ± 0.19	0.78 ± 0.14	*0.200*
5HT2B (*Htr2b*)	1.00 ± 0.30	1.13 ± 0.22	*0.598*	0.65 ± 0.31	1.1 ± 0.36	*0.066*	1.82 ± 0.64	1.73 ± 0.54	*0.712*	1.49 ± 0.60	1.16 ± 0.33	*0.154*
5HT7 (*Htr7*)	0.88 ± 0.61	1.12 ± 0.45	*0.621*	1.09 ± 0.76	0.84 ± 0.42	*0.246*	1.45 ± 0.73	1.09 ± 0.32	*0.114*	1.00. ± 0.41	0.80 ± 0.16	*0.380*
A2A (*Adora2a*)	1.00 ± 0.25	1.79 ± 0.28	**<*0.001***	0.76 ± 0.20	1.62 ± 0.25	**<*0.001***	1.37 ± 0.39	1.6 ± 0.38	**<*0.001***	1.36 ± 0.39	1.07 ± 0.33	*0.696*
PKCC δ (*Pkcd*)	1.00 ± 0.28	1.47 ± 0.40	*0.041*	0.62 ± 0.03	1.57 ± 0.52	**<*0.001***	1.6 ± 0.69	1.89 ± 0.35	*0.218*	1.28 ± 0.13	0.8 ± 0.17	*0.037*
p47phox (*Ncf1*)	1.00 ± 0.25	1.44 ± 0.27	*0.033*	0.80 ± 0.23	1.12 ± 0.13	*0.123*	1.80 ± 0.59	1.94 ± 0.55	*0.145*	1.40 ± 0.44	1.35 ± 0.34	*0.961*
gp91phox (*Cybb*)	1.00 ± 0.25	1.2 ± 0.70	*0.125*	0.77 ± 0.33	1.01 ± 0.47	*0.275*	1.08 ± 0.47	1.8 ± 0.66	*0.532*	1.29 ± 0.51	1.45 ± 0.44	*0.408*
phosphodiesterase 4B (*Pde4b*)	1.00 ± 0.23	1.42 ± 0.19	**<*0.001***	0.73 ± 0.16	1.09 ± 0.14	***0.001***	1.07 ± 0.22	1.07 ± 0.22	*0.077*	0.87 ± 0.15	0.78 ± 0.16	*0.401*
p38MapKinase β subunit (*Mapk11*)	1.08 ± 1.17	1.86 ± 0.37	**<*0.001***	0.66 ± 1.39	1.36 ± 0.24	**<*0.001***	1.30 ± 0.36	1.46 ± 0.40	*0.218*	0.91 ± 0.28	1.15 ± 0.32	*0.065*
p38MapKinase α subunit (*Mapk14*)	1.04 ± 1.24	1.68 ± 0.45	**<*0.001***	0.93 ± 1.24	1.24 ± 0.31	*0.310*	1.10 ± 0.22	1.38 ± 0.30	*0.088*	1.14 ± 0.51	1.07 ± 0.26	*0.908*
PKA Regulatory Subunit (*Prkaa1*)	1.00 ± 0.21	1.60 ± 0.39	**<*0.001***	0.86 ± 0.17	1.33 ± 0.22	***0.004***	1.12 ± 0.29	1.50 ± 0.37	***0.010***	1.02 ± 0.07	1.15 ± 0.11	*0.367*
PKA Catalytic Subunit (*Prkar1a*)	1.00 ± 0.11	1.31 ± 0.17	*0.016*	0.95 ± 0.37	1.14 ± 0.13	*0.133*	0.85 ± 0.22	1.33. ± 0.31	**<*0.001***	0.80 ± 0.19	1.13 ± 0.24	***0.006***
Fractalkine (*Cx3cl1*)	0.96 ± 0.53	1.54 ± 0.58	*0.027*	0.63 ± 0.20	1.38 ± 0.80	***0.004***	1.40 ± 0.35	1.87 ± 0.35	*0.082*	1.52 ± 0.21	0.85 ± 0.18	***0.005***
BDNF (*Bdnf*)	1.00 ± 0.33	1.12 ± 0.16	*0.465*	0.70 ± 0.39	1.27 ± 0.40	**<*0.001***	0.77 ± 0.34	1.07 ± 0.20	*0.052*	0.71 ± 0.28	0.97 ± 0.23	*0.085*
EPAC (*Epac1*)	1.00 ± 0.11	1.925 ± 0.43	**<*0.001***	0.78 ± 0.34	1.64 ± 0.44	**<*0.001***	0.95 ± 0.29	1.44 ± 0.31	***0.009***	1.31 ± 0.49	1.32 ± 0.11	*0.961*

**Table 5:** Effect of daily acute intermittent hypoxia (dAIH) on pro-plasticity gene expression. Significance p ≤ 0.01 (Bonferroni Correction).

Protein encoded (*gene*)	Younger male			Younger female			Middle aged male			Middle aged female		
	Sham	AIH	*p*	Sham	AIH	*p*	Sham	AIH	*p*	Sham	AIH	*p*
5HT2A (*Htr2a*)	1.00 ± 0.44	0.63 ± 0.17	***0.010***	0.89 ± 0.26	0.90 ± 0.15	*0.953*	0.85 ± 0.26	0.62 ± 0.19	*0.065*	1.02 ± 0.23	0.78 ± 0.14	*0.051*
5HT2B (*Htr2b*)	1.00 ± 0.30	0.65 ± 0.31	*0.183*	1.13 ± 0.22	1.1 ± 0.36	*0.916*	1.82 ± 0.64	1.49 ± 0.60	*0.146*	1.73 ± 0.54	1.16 ± 0.33	*0.014*
5HT7 (*Htr7*)	0.88 ± 0.61	1.09 ± 0.76	*0.665*	1.12 ± 0.45	0.84 ± 0.42	*0.198*	1.45 ± 0.73	1.00. ± 0.41	*0.057*	1.09 ± 0.32	0.80 ± 0.16	*0.189*
A2A (*Adora2a*)	1.00 ± 0.25	0.76 ± 0.20	*0.210*	1.79 ± 0.28	1.62 ± 0.25	*0.292*	1.37 ± 0.39	1.36 ± 0.39	*0.988*	1.6 ± 0.38	1.07 ± 0.33	***0.000***
PKCC δ (*Pkcd*)	1.00 ± 0.28	0.62 ± 0.025	*0.121*	1.47 ± 0.40	1.57 ± 0.52	*0.634*	1.6 ± 0.69	1.28 ± 0.13	*0.109*	1.89 ± 0.35	0.8 ± 0.17	***0.000***
p47phox (*Ncf1*)	1.00 ± 0.25	0.80 ± 0.23	*0.358*	1.44 ± 0.27	1.12 ± 0.13	*0.089*	1.80 ± 0.59	1.40 ± 0.44	*0.060*	1.94 ± 0.55	1.35 ± 0.34	***0.002***
gp91phox (*Cybb*)	1.00 ± 0.25	0.77 ± 0.33	*0.368*	1.2 ± 0.70	1.01 ± 0.47	*0.129*	1.08 ± 0.47	1.29 ± 0.51	*0.172*	1.8 ± 0.66	1.45 ± 0.44	*0.244*
phosphodiesterase 4B (*Pde4b*)	1.00 ± 0.25	0.73 ± 0.07	*0.019*	1.42 ± 0.19	0.94 ± 0.14	***0.001***	0.089 ± 0.08	0.87 ± 0.16	*0.814*	1.07 ± 0.24	0.78 ± 0.17	***0.006***
p38MapKinase β subunit (*Mapk11*)	1.08 ± 1.17	0.66 ± 1.39	*0.184*	1.86 ± 0.37	1.36 ± 0.24	***0.003***	1.30 ± 0.36	0.91 ± 0.28	*0.019*	1.46 ± 0.40	1.15 ± 0.32	*0.079*
p38MapKinase α subunit (*Mapk14*)	1.04 ± 1.24	0.93 ± 1.24	*0.824*	1.68 ± 0.45	1.24 ± 0.31	*0.014*	1.10 ± 0.22	1.14 ± 0.51	*0.800*	1.38 ± 0.30	1.07 ± 0.26	*0.115*
PKA Regulatory Subunit (*Prkaa1*)	1.00 ± 0.21	0.86 ± 0.17	*0.394*	1.60 ± 0.39	1.33 ± 0.22	*0.069*	1.12 ± 0.29	1.02 ± 0.07	*0.491*	1.50 ± 0.37	1.15 ± 0.11	*0.018*
PKA Catalytic Subunit (*Prkar1a*)	1.00 ± 0.11	0.95 ± 0.37	*0.717*	1.31 ± 0.17	1.14 ± 0.13	*0.152*	0.85 ± 0.22	0.80 ± 0.19	*0.688*	1.33. ± 0.31	1.13 ± 0.24	*0.084*
Fractalkine (*Cx3cl1*)	0.96 ± 0.53	0.63 ± 0.20	*0.180*	1.54 ± 0.58	1.38 ± 0.80	*0.462*	1.40 ± 0.35	1.52 ± 0.21	*0.956*	1.87 ± 0.35	0.85 ± 0.18	***0.000***
BDNF (*Bdnf*)	1.00 ± 0.33	0.70 ± 0.39	*0.085*	1.12 ± 0.16	1.27 ± 0.40	*0.309*	0.77 ± 0.34	0.71 ± 0.28	*0.672*	1.07 ± 0.20	0.97 ± 0.23	*0.509*
EPAC (*Epac1*)	1.00 ± 0.11	0.78 ± 0.34	*0.295*	1.925 ± 0.43	1.64 ± 0.44	*0.124*	0.95 ± 0.29	1.31 ± 0.49	*0.049*	1.44 ± 0.31	1.32 ± 0.11	*0.521*

**Table 6:** Serum estradiol level on daily acute intermittent hypoxia (dAIH) induced gene expression changes. Significance ≤0.01.

Protein encoded (*gene*)	R^2^		Diff in slope
	Sham	dAIH	*p value*
5HT2A (*Htr2a*)	0	0	*0.634*
5HT2B (*Htr2b*)	0.03	0	*0.521*
5HT7 (*Htr7*)	0.03	0.01	*0.720*
A2A (*Adora2a*)	0.16	0.02	*0.276*
PKCC δ (*Pkcd*)	0	0	*0.706*
p47phox (*Ncf1*)	0	0.08	*0.342*
gp91phox (*Cybb*)	0	0.02	*0.352*
phosphodiesterase 4B (*Pde4b*)	0.16	0.02	*0.030*
p38MapKinase β subunit (*Mapk11*)	0.2	0	*0.082*
p38MapKinase α subunit (*Mapk14*)	0.31	0.09	***0.001***
PKA Regulatory Subunit (*Prkaa1*)	0.2	0.02	*0.157*
PKA Catalytic Subunit (*Prkar1a*)	0.18	0	*0.077*
Fractalkine (*Cx3cl1*)	0.016	0.01	*0.462*
BDNF (*Bdnf*)	0.01	0.11	*0.531*
EPAC (*Epac1*)	0.29	0.03	*0.121*

## Data Availability

Data will be made available on request.
